# Cyclodextrin-Based Metal–Organic Framework as an Application Platform for Bioactive Ruthenium(III) Complexes

**DOI:** 10.1021/acs.inorgchem.5c00813

**Published:** 2025-05-23

**Authors:** Mahya Asgharian Marzabad, Sára Kollárová, Fangfang Pan, Martin Novák, Jan Kuta, Kari Rissanen, Pavel Babica, Radek Marek, Ondřej Jurček

**Affiliations:** † Department of Chemistry, Faculty of Science, 37748 Masaryk University, Kamenice 5, 62500 Brno, Czechia; ‡ Department of Natural Drugs, Faculty of Pharmacy, Masaryk University, 61200 Brno, Czechia; § CEITEC - Central European Institute of Technology, Masaryk University, Kamenice 5, 62500 Brno, Czechia; ∥ College of Chemistry, 12446 Central China Normal University, 152 Luoyu Road, Wuhan 430079, China; ⊥ RECETOX, Faculty of Science, Masaryk University, Kotlářská 2, 61137 Brno, Czechia; # Department of Chemistry, University of Jyvaskyla, P.O. Box 35, 40014 Jyväskylä, Finland

## Abstract

Ruthenium­(III) complexes are promising anticancer metallodrugs because of their antimetastatic (migrastatic) potential and significantly lower host toxicity than generally used platinum metallodrugs. On the other hand, the ruthenium­(III) complexes generally show low solubility and stability in an aqueous environment but exhibit some toxicity associated with unspecific delivery. For these reasons, numerous ongoing studies deal with their encapsulation into various delivery systems to maximize their therapeutic efficacy. One of these systems can also be crystals of nontoxic metal–organic frameworks (MOFs). In this work, we studied incorporation of a bioactive ruthenium­(III) complex (RuC) inside MOFs derived from γ-cyclodextrin (γ-CD) and biocompatible potassium ions, forming CD-MOF-1. Viability studies *in vitro* were carried out using spheroids of human hepatoblastoma cell line HepG2. These studies revealed that the RuC-CD-MOF-1 system provides effective cancer cell suppression through slow gradual release over a longer period (>10 days) while reducing acute cytotoxic effects associated with naked RuC. This combination was defined for further development and optimization as a drug-delivery platform for metallodrugs.

## Graphical abstract

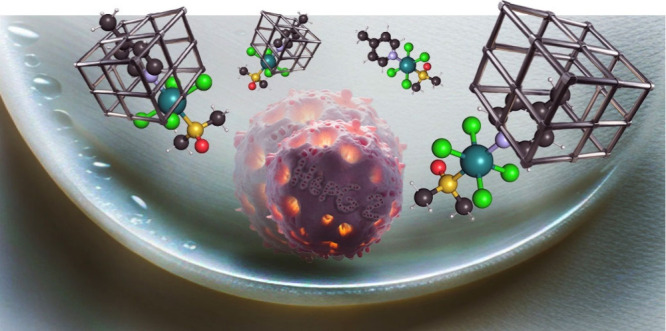

## Introduction

With the rapid advancement of materials chemistry, much effort has been directed to the development of innovative micro- and nanoplatforms for controlled drug release with the goal of maximizing therapeutic efficacy while minimizing side effects.^1^ Metal–organic frameworks (MOFs) composed of organic ligands and metal ions (or metal clusters) linked together by coordination bonds forming one-, two-, or three-dimensional, highly porous, and crystalline materials are one example of such systems.^2^ Aside from a wide range of applications such as gas adsorption and storage,^3,4^ molecular recognition,^5^ ion exchange,^6^ sensing,^7^ catalysis,^8^ or separation,^9^ MOFs have also found applications in drug delivery.^10,11^ Transition metals,^12^ p-block elements,^13^ alkali metals,^14^ alkaline earth metals,^15^ lanthanides,^16^ and actinides^17^ are among the metal ions employed in the production of MOFs. Similarly, the organic linkers are diverse enough to contain functional groups such as carboxylate, phosphonate, sulfonate, pyridyl, imidazolate, or azolate.^3^ However, there are only a limited number of organic metal salts and organic linkers that are biocompatible and nontoxic at therapeutic doses.^18^

MOFs in comparison with other drug nanocarriers such as organic polymers,^19^ quantum dots,^20^ or inorganic nanoparticles^21^ demonstrated additional advantages such as higher drug loading capacity, lower toxicity, slower release, and fewer side effects.^22–24^ A wide range of cargos can be efficiently loaded in MOFs, e.g., small medicinal molecules,^25,26^ peptides, or even DNA/RNA.^27,28^ So far, reports describing the loading of bioactive coordination complexes inside MOFs are rather scarce.^29^

Most MOFs are prepared by heating a solution of a metal salt and organic linker (solvothermal technique). Aside from the commonly used solvothermal methods, there are numerous alternative options, such as vapor diffusion, microwave-assisted, electrochemical, mechanochemical, or sonochemical synthesis.^30^ To decrease the cost of MOF synthesis, numerous green synthesis techniques for MOFs based on biocompatible metal ions such as Ca­(II), K­(I), and Ti­(III) have been proposed.^31,32^ Similarly, biologically suitable linkers such as peptides,^33^ polysaccharides,^34^ amino acids,^35^ or cyclodextrins (CDs)^36,37^ or their derivatives^38^ have been employed to reduce the biological risks and cost of MOFs. There are three main types of CDs generally recognized: α-CD (built of six glucose units interconnected by α-1,4-glycosidic bonds), β-CD (seven glucose units), and γ-CD (having eight units). The water-soluble CDs readily form inclusion complexes with various hydrophobic molecules and drugs.^39,40^ Additionally, because of CD’s coordination ability and also its *C*_4_ symmetry, the γ-CD has been utilized in the green preparation of biocompatible and nontoxic MOFs (CD-MOFs) using alkali and alkaline earth metal ions. The CD-MOFs are formed via diffusion of ethanol into their aqueous solution, where CD-MOF-1 is generally formed using γ-CD and potassium hydroxide.^26,36,37,41^ CD-MOFs have shown some advantageous features in the production of oral medical formulations.^25,26,42,43^

In general, the inclusion complexes of CDs with organic drug molecules are well-established pharmaceutical forms used clinically.^44,45^ To date, there is not any clinical example of the use of CDs as carriers of bioactive coordination complexes. Nevertheless, the potential of CDs as a single molecular host system for the uptake of metallodrugs has been shown,^46,47^ also, quite recently for ruthenium­(III) complexes.^48,49^ Ruthenium-based anticancer drugs are regarded to have a great promise because of their migrastatic (antimetastatic) activities and low host toxicity.^50^ Their selective antimetastatic activity was identified for *cis*- and *trans*-[RuCl_2_(DMSO)_4_] in the early 1980s, while the most significant progress in ruthenium anticancer activity was made with NAMI (Na­[*trans*-RuCl_4_(DMSO-S)­(ImH)]) (where ImH is imidazole) and NAMI-A ([ImH_2_]­[*trans*-RuCl_4_(DMSO-S)­(ImH)]) compounds.^51^ KP1019 has also shown significant activity against primary tumors, especially against colorectal cancer. In general, the ruthenium metallodrugs show fewer side effects than cisplatin, and they also show activity against cisplatin-resistant cancer. In contrary, given the poor stability, low water solubility, and yet some toxicity associated with the unspecific delivery of ruthenium metallodrugs, the development of effective carrier systems can improve their pharmacological profile.^29^

This study focuses on the synthesis and stability assessment of a model complex consisting of ruthenium­(III) coordinated to 4-methylpyridine (4-picoline and 4-MePy) and DMSO in axial positions and four chlorides in equatorial ones (RuC (Scheme 1)), [RuCl_4_(4-MePy)­(DMSO)],^52^ and its adsorption into CD-MOF-1. The system was subsequently studied in a viability study *in vitro* using spheroids of human hepatoblastoma cell line HepG2 showing a potential of such metallodrug formulation for increasing its therapeutic effect.

**Scheme 1:**
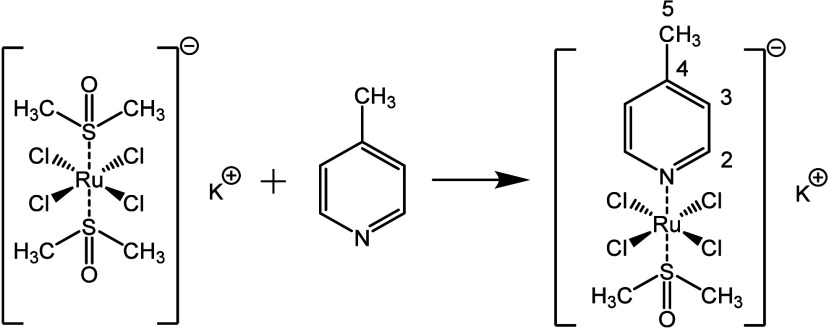
Preparation of RuC and Carbon Numbering of the 4-Methylpyridine Ligand

## Experimental Section

### Synthesis of RuC and CD-MOF-1

The synthesis of RuC was previously described (Scheme 1),^52,53^ and a slightly modified procedure used is described in detail in section 2 of the Supporting Information (Figures S1 and S2). CD-MOF-1 was prepared by vapor diffusion according to a previously published procedure.^26^

### HepG2 Cell Cultivation, Spheroid Preparation, Exposure, and Toxicity Assessment

HepG2 cell cultivation and spheroid preparation and toxicity assays were performed following the literature.^54,55^ Further details are reported in section 6 of the Supporting Information.

## Results and Discussion

### Encapsulation of RuC into CD-MOF-1

The CD-MOF-1 crystals were prepared using γ-CD and KOH.^26^ As such, the crystals formed possessed a highly alkaline inner environment that can easily lead to decomposition of sensitive guest molecules. Indeed, ligand replacement was observed in adsorption experiments with untreated (as-synthesized) but activated crystals of CD-MOF-1 using an analogous cheaper Ru­(III) complex [RuCl_4_(Py)­(DMSO)] as a guest. Interestingly, in these experiments, we have found that the adsorption of [RuCl_4_(Py)­(DMSO)] or its decomposition products can trigger a crystal-to-crystal transformation, and the cubic structure of CD-MOF-1 transforms into a two-dimensional columnar network (Figure 1; crystal description in section 3.1 of the Supporting Information) (a similar transformation of CD-MOF-2 derived from γ-CD and RbOH was observed as shown in Figure S3). Transformation also proceeds just by using metal salts, such as PdCl_2_, PtCl_4_, or RhCl_3_. Considering these conditions, the inclusion of RuC into crystals during MOF synthesis is not viable. Instead, we have investigated a pathway of RuC postsynthetic adsorption into pretreated and activated crystals of CD-MOF.

**Figure 1:**
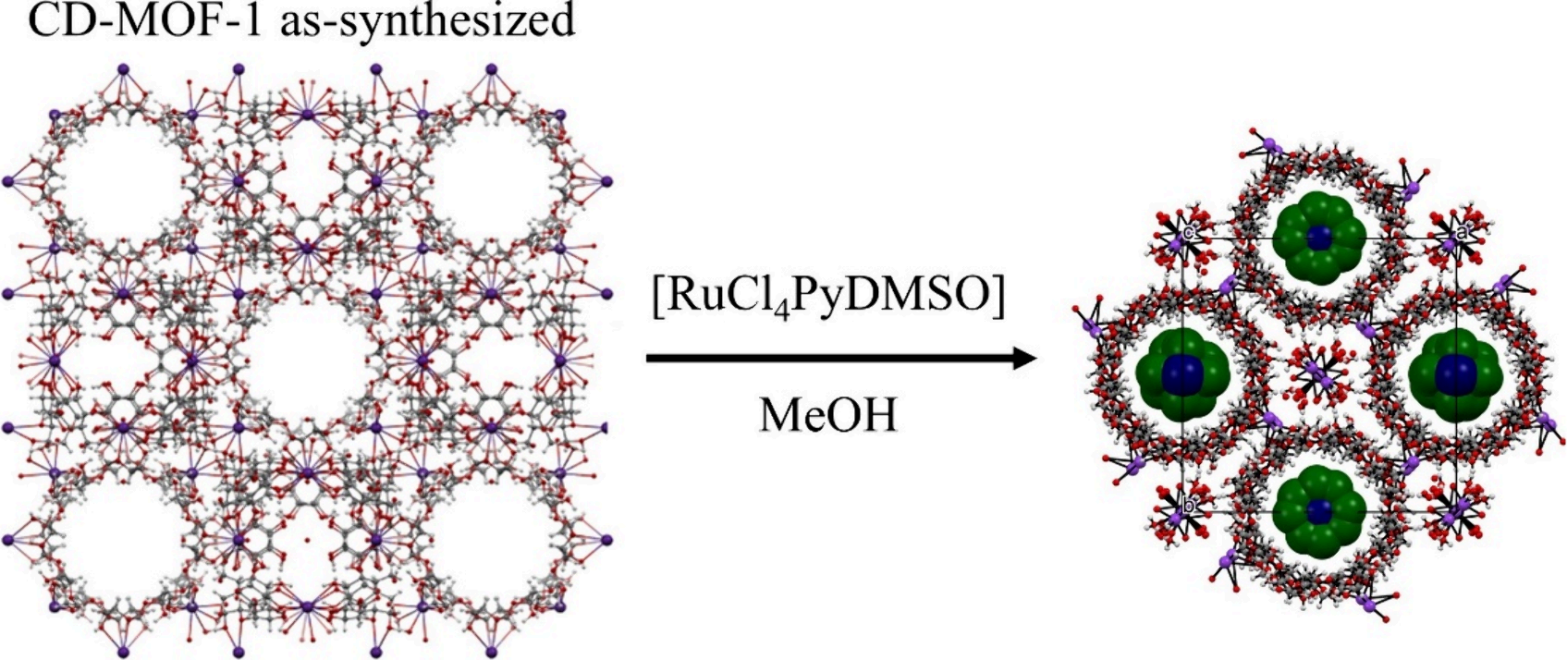
Crystal-to-crystal transformation of the cubic crystal structure of CD-MOF-1 (K^+^-based CD-MOF) into a columnar structure where the pores contain decomposition products of [RuCl_4_(Py)­(DMSO)] (RuCl_4_ could be assigned). CCDC 2419920.

The crystals of CD-MOF-1 as synthesized were neutralized by suspending in an ethanolic solution of hydrochloric acid (1 M, 500 μL) for 24 h at ambient temperature. Subsequently, the crystals were filtered off and washed with ethanol (3 × 1 mL). The colorless cubic crystals were finally dried under a high vacuum for 24 h. The final pH of the processed crystals was 6. The stability of the crystal structure of CD-MOF-1 upon processing was confirmed by comparison with the as-synthesized CD-MOF-1 sample using powder X-ray diffraction (PXRD) analysis (Figures S4). The morphology of the crystals processed was checked by scanning electron microscopy (SEM) (Figure S5a).

In general, the Ru­(III) complexes are unstable, undergoing relatively swift hydrolysis/solvolysis; therefore, stability experiments were carried out to find the most convenient conditions for a potentially long period of their occlusion into the crystals of CD-MOF-1 (section 5 of the Supporting Information). Moreover, considering CD-MOF-1 and RuC solubility, the choice of the solvent system and pH plays an important role. Based on these, methanol and DMSO (or their deuterated forms easing analysis by ^1^H NMR) were studied with varying contents of HCl (DCl). Finally, RuC has shown sufficient solubility and stability in a DMSO-*d*_6_ solution at pH 4 (Figure 2). This system also does not represent any significant harm to crystals of CD-MOF-1 and additionally maintain the lower pH of the potentially remaining basic inner environment of CD-MOF-1, further stabilizing RuC during adsorption. In general, the characterization of the stability of Ru­(III) complexes is a rather challenging task as there can be a great number of species formed, and as such, establishing an equilibrium is a complex process. To add to that, there are just a very few methods providing detailed information about ligand exchange, e.g., electron paramagnetic resonance spectroscopy (EPR)^53^ or nuclear magnetic resonance spectroscopy (NMR), where the NMR method is generally easily available and used more often.^52,56,57^ Figure 2 shows characteristic ^1^H NMR signals of RuC, similar to those as we previously studied.^52,56,57^ Whereas the NMR signals of hydrogens at C2, the closest to the Ru­(III) center, thus being affected the most by the hyperfine interaction,^57^ are not observable in the ^1^H NMR spectrum because of paramagnetic relaxation, the NMR signals of hydrogens at C3 and C5 are broad and show negative NMR shifts, −2 and −3 ppm, respectively. Similarly, from the other side of the vertical axle, the methyl signals of dimethyl sulfoxide coordinated directly to the Ru­(III) center show a significant ^1^H NMR shift at −13 ppm and even larger relaxation broadening. The time-dependent NMR study of RuC at pH 4 shows the appearance of another set of H2 and H3 signals corresponding to free 4-picoline after 24 h (Figure 2), signing a negligible ligand release, which further moderately increases over time. Unlike under other conditions studied (see section 5 of the Supporting Information), there are no other significant side products observed.

**Figure 2:**
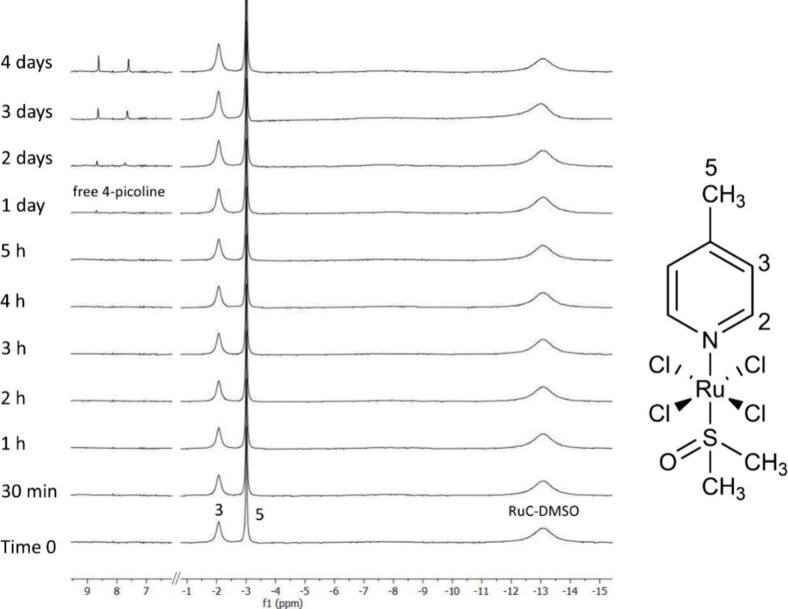
^1^H NMR spectra of RuC measured at different times (0–4 days) at pH 4 (DMSO-*d*_6_, 700 MHz, 298.2 K).

In the next step, colorless CD-MOF-1 crystals (14.0 mg) were placed in an orange DMSO solution of RuC (200 μL, 86 mM, pH 4). The suspension was left standing in the dark for 3 days, over which the colorless cubic crystals turned orange. The crystals were collected and washed with DMSO (3 × 1 mL) and ethanol (3 × 1 mL) and finally dried under high vacuum overnight (Figure S5b). The content of ruthenium inside the crystals was determined using inductively coupled plasma mass spectrometry (ICP-MS) resulting in a value of 1.82 wt %, which corresponds to 8.1 wt % RuC in RuC-CD-MOF-1 crystals. The content of RuC was also determined using ^1^H NMR spectroscopy on dissolved RuC-CD-MOF-1 crystals (section 6.1 of the Supporting Information and Figure S14) or indirectly using UV–vis spectroscopic measurement of the residual RuC concentration in the mother liquor (section 6.2 of the Supporting Information and Figure S15); nevertheless, both are burdened by methodical errors as further discussed in the Supporting Information. The crystals of RuC-CD-MOF-1 were ground into microparticulate powder and used in biological studies (Figure S5c).

### Biological Studies

We chose three-dimensional (3D) HepG2 spheroids for our initial toxicity study, as they represent a well-established and characterized *in vitro* model commonly used in liver cancer research.^58,59^ These spheroids effectively mimic the physiological tumor microenvironment, including structural organization, cell–cell and cell–extracellular matrix (ECM) interactions, central hypoxia, and oxygen and nutrient gradients.^58–61^ Notably, HepG2 cells have been previously utilized in anticancer *in vitro* studies involving ruthenium complexes,^62^ including applications in spheroid models.^63–65^

In this study, we investigated the effects of γ-cyclodextrin (γ-CD), CD-MOF-1, the free Ru­(III) complex (naked RuC), and RuC encapsulated within CD-MOF-1 (RuC-CD-MOF-1) on the viability and growth dynamics of HepG2 spheroids over a 14-day period. The concentrations of RuC-CD-MOF-1 samples refer to the concentrations of RuC in the test system, calculated from the amount of RuC incorporated inside CD-MOF-1, as determined using the ICP-MS method. Throughout all concentrations, HepG2 spheroids treated with γ-CD and CD-MOF-1 alone exhibited no significant changes in the spheroid area or growth patterns compared to the solvent control that maintained continuous growth during the experiment (Figures S16–S18). Moreover, the ATP levels in the spheroids treated with γ-CD and CD-MOF-1 were comparable to those in the solvent control spheroids, indicating that the carriers did not affect the number of viable cells in the spheroids (Figure 3). This lack of cytotoxicity demonstrates that both γ-CD and CD-MOF-1 carriers are biocompatible, posing no inherent toxicity at the concentrations tested.

**Figure 3:**
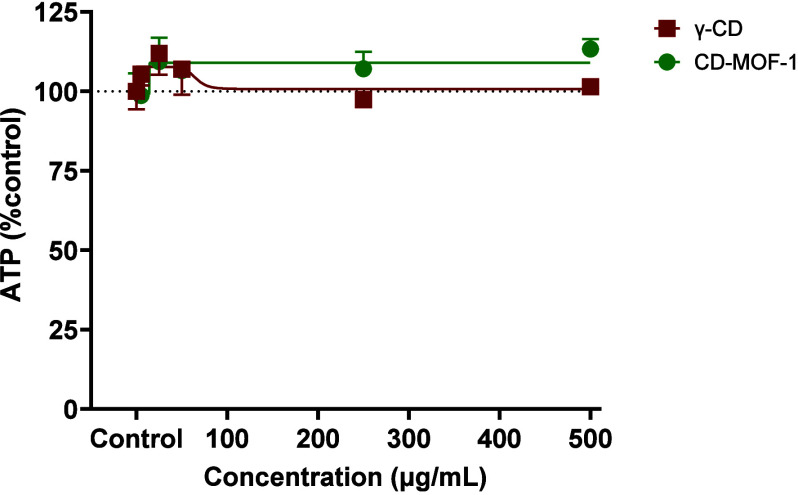
Spheroid viability after 14 days of exposure to γ-CD and CD-MOF-1. Spheroid viability was assessed using an ATP assay, with results expressed as a percentage of the ATP level relative to the control. Data represent the mean ± SEM from independently repeated experiments (*n* = 3). No treatment was significantly different from the solvent control (ANOVA; *P* < 0.05).

In contrast, spheroids exposed to naked RuC showed dose-dependent inhibition of growth, particularly at 400 and 500 μg/mL (Figure 4a and Figures S19a and S20). Initially, these spheroids grew normally until day 4, similar to the case for the control. Afterward, a marked cytotoxic response emerged, characterized by a significant inhibition of growth. At 400 μg/mL, the spheroid size stagnated from day 4 onward, while the longer exposures to the highest concentration of RuC even slightly reduced the spheroid size compared to day 4. These results indicate a sustained, potent cytotoxic effect from the naked RuC. On the other hand, RuC at concentrations of 100–200 μg/mL rather increased spheroid size after day 7, while only a nonsignificant reduction of spheroid size was caused by 300 μg/mL on day 14. However, a concentration-dependent reduction in ATP levels induced by RuC was observed starting at 100–200 μg/mL, resulting in an approximate 15% decrease. At 300 μg/mL, cell viability was reduced to around 60% of the control, while the two highest concentrations caused a statistically significant cytotoxic effect, reducing viability to 25–35% of the control (Figure 4). These results highlight the potent cytotoxicity of RuC against HepG2 cells in its free form, where its rapid availability leads to substantial cell damage. Treatment with RuC encapsulated in CD-MOF-1 resulted in a notably different growth pattern (Figure 3b and Figures S19b and S21). The spheroids treated with 100–300 μg/mL exhibited a slight but significant and progressing growth reduction after day 7, indicating that CD-MOF-1 encapsulation delayed the cytotoxic impact of RuC. This significant reduction in spheroid size was also accompanied by a 15–30% decrease in ATP levels, although this change was not statistically significant (Figure 5). In contrast, spheroids exposed to encapsulated RuC at concentrations of 400–500 μg/mL exhibited a significant increase in size from day 4, peaking on day 11, followed by a reduction in size by day 14 (Figure 4b). This growth pattern was accompanied by a 30–40% decrease in ATP levels after 14 days of exposure (Figure 5).

**Figure 4:**
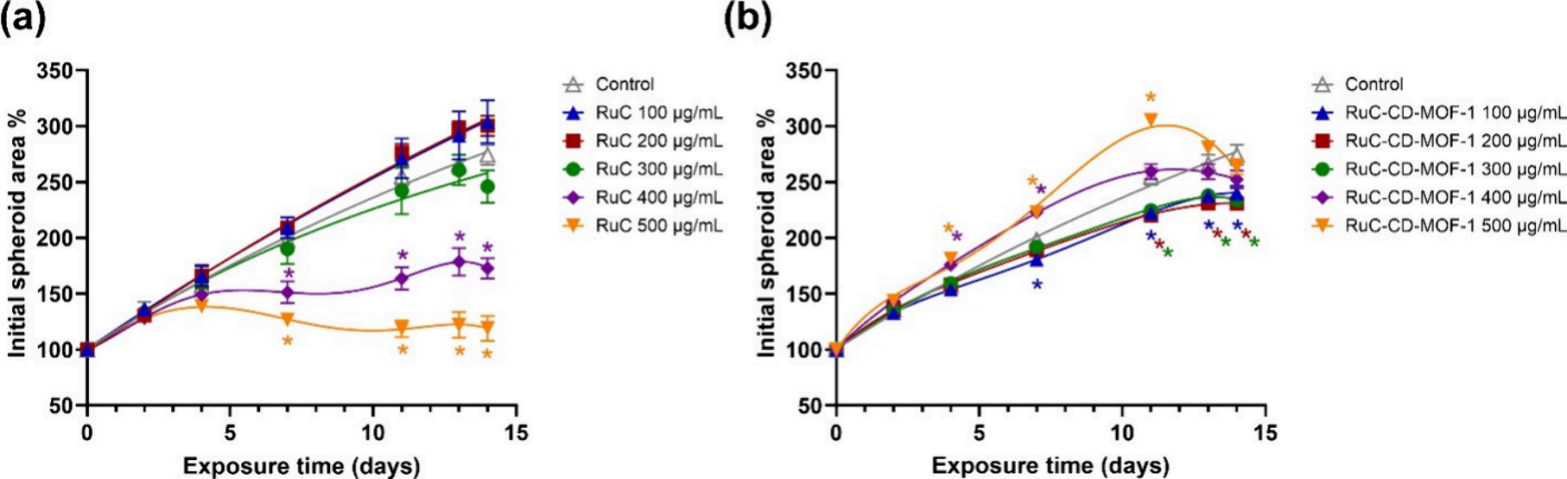
Monitoring of the HepG2 spheroid growth during a two-week exposure. Plots are based on the relative change in the projected spheroid area over time under a given treatment, compared to the initial area at the start of the exposure (day 0, set as 100%). Samples of (a) RuC and (b) RuC-CD-MOF-1 (the concentrations refer to the encapsulated RuC in the test system). Data represent the mean ± SEM from independently repeated experiments (*n* = 3). Asterisks represent treatments significantly different from the solvent control at the given time point (ANOVA; *P* < 0.05).

**Figure 5:**
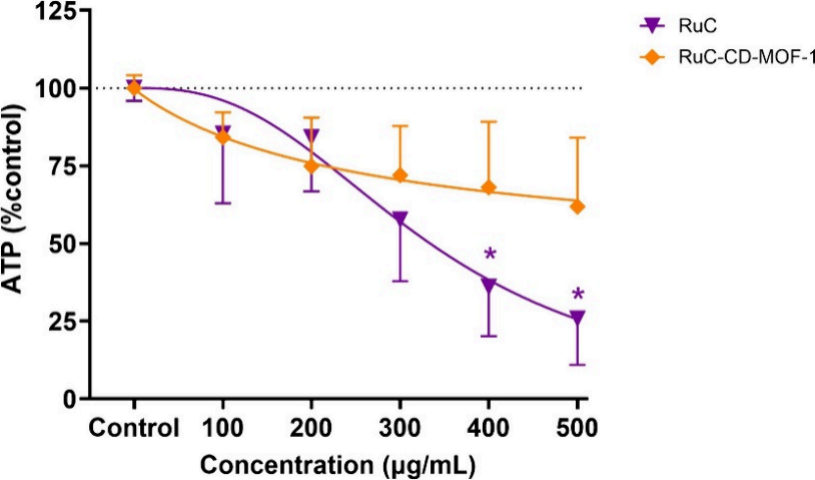
Spheroid viability after 14 days of exposure to RuC and RuC-CD-MOF-1 (the concentrations refer to the encapsulated RuC in the test system). Spheroid viability was assessed using an ATP assay, with results expressed as a percentage of the ATP level relative to the control. Data represent the mean ± SEM from independently repeated experiments (*n* = 3). Asterisks represent treatments significantly different from the solvent control (ANOVA; *P* < 0.05).

Overall, encapsulated RuC affected spheroid size and viability, albeit with milder and delayed effects compared to those of naked RuC. This could be attributed to the controlled release of RuC from CD-MOF-1, which allowed spheroids to continue growing initially, with cytotoxic effects emerging later. These findings suggest that CD-MOF-1 encapsulation facilitates a more gradual and sustained cytotoxic response over the treatment period, mitigating the more immediate and stronger cytotoxicity of the free Ru­(III) complex.

Interestingly, the observed increase in spheroid size despite reduced ATP levels could be explained by several factors. (1) Cytotoxic stress responses may alter cell metabolism, triggering compensatory mechanisms such as increased cell size or proliferation.^66,67^ (2) Necrotic core formation within spheroids may allow outer layers to continue expanding even as inner regions lose viability.^68^ (3) Disruptions in cell cycle regulation can lead to cell enlargement or delayed apoptosis, contributing to spheroid growth.^69^ (4) Heterogeneous cell populations within spheroids may contain subpopulations resistant to RuC, supporting continued growth.^70,71^ (5) Increased extracellular matrix production or remodeling in response to cellular stress may also contribute to overall spheroid expansion.^72^

The comparative cytotoxic profiles of naked and encapsulated RuC highlight the substantial influence of encapsulation on the release kinetics and therapeutic efficacy in HepG2 spheroids. The more rapid cytotoxic response observed with the naked Ru­(III) complex is likely due to its swift and unrestricted interaction with the spheroids that induces cellular stress responses and cell damage. This can affect the function of mitochondria and cell energy metabolism, leading to a slowdown of cellular division and decreasing the ATP levels. Ru­(III) complexes can potentially act via more significant mechanisms like DNA interaction and damage, similarly generating oxidative stress, mitochondrial damage, or endoplasmic reticulum (ER) stress.^73,74^ This could lead to a reduction in spheroid area during this initial phase due to impaired proliferation caused by cell cycle arrest resulting from DNA damage. Additionally, disruption of energy metabolism due to mitochondrial damage, along with the induction of cell death via mechanisms such as apoptosis or autophagic processes triggered by DNA or mitochondrial damage or ER stress,^75^ may further contribute to the observed reduction in spheroid size. Such immediate cytotoxicity, although effective at curtailing growth, may inadvertently drive adaptive stress responses that could facilitate the emergence of aggressive, potentially resistant cells.

In contrast, RuC-CD-MOF-1 exhibited notably less steep concentration–response curves, indicating the milder and gradual effects of RuC. The highly porous structure of CD-MOF-1 likely plays a critical role here by slowly releasing RuC, unlike in the case of the more intense effect observed with the application of its free form. This might be crucial for therapeutic applications in which minimizing acute toxicity to surrounding healthy tissues is of paramount importance. The differential effects on cell viability between encapsulated and free forms suggest that CD-MOF-1 encapsulation can fine-tune the therapeutic index of Ru­(III)-based complexes. By providing a sustained therapeutic concentration, encapsulation could extend the therapeutic window, allowing prolonged cytotoxic action against cancer cells while reducing the likelihood of excessive and off-target toxicity. This controlled release strategy could be particularly advantageous for treating aggressive tumors, where gradual, consistent therapeutic pressure may prevent rapid adaptation and resistance, ensuring effective treatment over an extended period.

There are two possible explanations for the decrease in ATP concentration. On one hand, the cytotoxicity of RuC is demonstrated through, e.g., increased oxidative stress, which affects the function of mitochondria and cell energy metabolism, leading to slowdown of cellular division: a smaller size of spheroids and lower ATP levels. Alternatively, RuC causes more significant cellular/DNA damage, leading to increased cell death, which ultimately has a similar effect on the spheroid size and ATP concentration.

In summary, the encapsulation of Ru­(III) within CD-MOF-1 offers a promising approach to modulating its therapeutic efficacy. Particularly in oncological settings, the controlled release of RuC could delay acute cytotoxic effects, prevent excessive early toxicity, and provide sustained therapeutic pressure, thus potentially inhibiting both tumor growth and metastatic spread.^73^ These findings underscore the potential of CD-MOF-1 as a prospective drug-delivery system that modulates the pharmacokinetics of metal-based drugs.

## Conclusion

In conclusion, the encapsulation of the ruthenium­(III) complex (RuC) in CD-MOF-1 demonstrates a promising approach to address long-standing challenges in the clinical translation of ruthenium-based anticancer therapies, such as stability, solubility, and toxicity. Through *in vitro* studies using 3D HepG2 spheroids, we observed that encapsulating RuC in CD-MOF-1 modulates the profile and kinetics of the cytotoxic effects of the complex, most likely due to a controlled release. The controlled release mechanism provided by CD-MOF-1 delayed the onset of cytotoxicity compared with free RuC, allowing for a sustained decrease in spheroid growth and viability with less steep concentration–response curves. This finding underscores the potential of CD-MOF-1 encapsulation to achieve effective cancer cell suppression while reducing acute cytotoxic effects associated with naked RuC, suggesting an improved therapeutic window and a more favorable safety profile. The delayed but steady cytotoxic action of the RuC-CD-MOF-1 formulation, marked by a gradual decrease in spheroid area and ATP levels from day 8 to 14, supports the potential for long-term tumor suppression. This controlled-delivery system may also reduce the risk of adverse side effects and systemic toxicity compared with the naked RuC, which showed a stronger cytotoxic response with a steeper profile, potentially increasing the risk of acute toxicity. Importantly, the capacity of the encapsulated RuC to maintain therapeutic pressure on HepG2 cells aligns with the need for controlled release strategies in cancer treatment, especially in metastatic contexts where sustained drug exposure may be beneficial. In summary, our findings support the idea that encapsulation of RuC in CD-MOF-1 provides a model for optimizing the balance between cytotoxicity and therapeutic efficacy in metallodrug development. The observed therapeutic benefit of RuC-CD-MOF-1 highlights the importance of considering controlled release mechanisms in advancing metallodrugs and suggests a path forward in designing MOF-based delivery systems to maximize anticancer effects while minimizing systemic toxicity. These features can be further improved and optimized through particle surface modifications and control over crystals’ morphology.

In future studies, we will aim to expand to a broader panel of liver cancer spheroids (e.g., Hep3B, Huh-7, PLC/PRF/5, and SNU-398)^59^ but also of other cancer types (e.g., colon, breast, lung, and pancreas) to enhance the robustness, specificity, and translational relevance of our findings by capturing the heterogeneity of tumors and their differential drug responses. Moreover, according to a common hypothesis, the Ru­(III) complexes are considered as prodrugs activated by reduction to Ru­(II).^76,77^ However, at the same time, the considered reduction is still a matter of ongoing discussion.^78^ Therefore, we also consider including an intracellular study of the mode of action of this application form.

## Supplementary Material



## Notes

Description of the general analytical methods used; synthesis, characterization, and stability study of the Ru­(III) complex; drug loading study and determination of the drug content; and HepG2 spheroid preparation, exposure, and toxicity assessment (PDF)
