# Supplementary material for: Cyclodextrin-Based Metal–Organic Framework as an Application Platform for Bioactive Ruthenium(III) Complexes
Source: Inorg Chem. 2025 May 23;64(22):10870–8. doi: 10.1021/acs.inorgchem.5c00813 (PMC12152939; doi:10.1021/acs.inorgchem.5c00813)
Supplement: Supplementary file 1 [file ic5c00813_si_001.pdf]

Supporting Information

Cyclodextrin-Based Metal-Organic Framework as Application Platform for Bioactive Ruthenium(III)-Complexes

Mahya Asgharian Marzabad,[1,2,3] Sára Kollárová,[1] Fangfang Pan,[4] Martin Novák,[1,3] Jan Kuta,[5] Kari Rissanen,[6] Pavel Babica,[5] Radek Marek,[1,3*] Ondřej Jurček[1,2,3*]*

[1] Department of Chemistry, Faculty of Science, Masaryk University, Kamenice 5, CZ-62500 Brno, Czechia

[2] Department of Natural Drugs, Faculty of Pharmacy, Masaryk University, 61200 Brno, Czechia

[3] CEITEC – Central European Institute of Technology, Masaryk University, Kamenice 5, CZ-62500 Brno, Czechia

[4] Chemical building of Central China Normal University, College of Chemistry, Central China Normal University, Wuhan city Hongshan District Luoyu Road No. 152, China

[5] RECETOX, Faculty of Science, Masaryk University, Kotlářská 2, 61137 Brno, Czechia

[6] Department of Chemistry, University of Jyvaskyla, P.O. Box 35, 40014 Jyväskylä, Finland

CONTENT

1. General methods 3
1.1 Materials 3
1.2 ^{1}H NMR spectroscopy 3
1.3 Electrospray ionization mass spectrometry (ESI-MS) 4
1.4 Single crystal X-ray diffraction (SCXRD) 4
1.5 Powder X-ray diffraction (PXRD) 4
1.6 UV-Vis spectroscopy 5
1.7 Inductively coupled plasma mass spectrometry (ICP-MS) 5
1.8 Scanning electron microscopy (SEM) 5
1.9 Microplate readers 6
2 Synthesis and characterization of Ru(III) complex (RuC) 6
3 Crystal-to-crystal transformation 9
3.1 Single crystal X-Ray diffraction (SC-XRD) 9
4 Stability and morphology of crystals of CD-MOF-1 10
4.1 Powder X-Ray diffraction (PXRD) 10
4.2 Crystal morphology 11
5 Stability studies of RuC 11
6 Drug loading studies 22
6.1 ^{1}H NMR spectroscopic study 22
6.2 UV-Vis spectroscopic study 23
7 HepG2 cell cultivation and spheroid preparation 24
8 HepG2 spheroid exposure and toxicity assessment 25
9 References 30

1. General methods

1.1 Materials

All chemicals were commercially available and were used without further purification. K^+[*trans*-$RuCl_4(DMSO)_2$], acetonitrile, acetone, 4-picoline, potassium hydroxide, γ-cyclodextrin were purchased from Merck. Human hepatoblastoma cells HepG2 (ATCC® HB-8065™) were purchased from LGC Standards (Łomianki, Poland). Minimum Essential Medium (MEM) with phenol red (Gibco Cat. No. 61100), MEM Non-Essential Amino Acids (Gibco Cat. No. 11140), sodium pyruvate, penicillin/streptomycin (P/S) and trypsin/EDTA were purchased from Thermo Fisher, Waltham, MA. Agarose (Cat. No. A9539), sodium bicarbonate and sodium chloride were purchased from Sigma-Aldrich, Prague, Czech Republic. Fetal bovine serum (FBS, Cat No. 1001/500) was purchased from Biosera, Nuaillé, France. Sterile 0.2 μm PES filters, and cell culture flasks were purchased from TPP, Trasadingen, Switzerland. CellTiter-Glo® 3D cell viability assay was purchased from Promega, Madison, WI. Black 96-well plates with transparent bottom (CELLSTAR®, Cat. No. 655090) were purchased from Greiner Bio One, Kremsmünster, Austria.

1.2 ^{1}H NMR spectroscopy

^{1}H NMR spectra were recorded on a Bruker Avance III HD 700, 500 or 300 MHz spectrometers at the 298.15 K temperature. All NMR spectra are referenced to the residual internal solvent signal of DMSO-d_6 (2.5 ppm) or CD_3OD (3.31 ppm). ^{1}H NMR spectral data are presented in the following order: chemical shift (δ) expressed in ppm, multiplicity and coupling constants in Hertz. Data were analyzed using Mestrenova Program (v1.13).

1.3 Electrospray ionization mass spectrometry (ESI-MS)

Mass spectrometric analysis of compounds synthesized was carried out on Ultraflextreme Bruker Daltonics machine. Additional details are shown in the title of spectrum.

1.4 Single crystal X-ray diffraction (SCXRD)

The single crystals of $RuCl_4$@CD-MOF-1 were obtained through crystal-to-crystal transformation as described in Supplementary section 3. Single-crystal X-ray diffraction data were collected using a dual-source Rigaku SuperNova Oxford diffractometer equipped with an Atlas detector using mirror-monochromated Cu-$K\alpha$ radiation (λ = 1.54184 Å). The data collection and reduction were carried out using the program CrysAlisPro.[1] Intensities were corrected for absorption using the Analytical numeric absorption correction method.[1] Structures were solved with direct methods (SHELXS)[2,3] and refined by full-matrix least squares on F^2 using OLEX2,[4] which utilizes the SHELXL-2015 module.[2,3]

1.5 Powder X-ray diffraction (PXRD)

The crystallinity and phase purity of samples were determined by PXRD. The samples were pressed on an aluminium slide, sample height was aligned with laser, and data were collected in the range $3° < 2\theta < 90°$. The diffraction data were collected on a RIGAKU SmartLab 3 kW diffractometer with a fine focus Cu sealed tube with graphite monochromated MoKα at 40 kV, 30 mA.

1.6 UV-Vis spectroscopy

UV-visible absorption spectra were recorded using Agilent Cary 60 spectrophotometer (USA) with customized sample chamber (which can be fitted with cuvettes and solid samples) in the range of 200-800 nm. The UV-Vis spectra were recorded at room temperature using 10 mm quartz cuvette.

1.7 Inductively coupled plasma mass spectrometry (ICP-MS)

Content of Ru in RuC-CD-MOF-1 was determined by inductively coupled plasma mass spectrometry (Agilent 7700x ICP-MS, Agilent Technologies) after sample dissolution in mixture of hydrochloric and nitric acid. Briefly, 1.57 mg of RuC-CD-MOF-1 was mixed with 0.3 ml of 65% nitric acid (Suprapur, Merck), 0.10 ml of 36% hydrochloric acid (Suprapur, Merck) in PP falcon type tube and subsequently diluted to 10 ml with Milli-Q type 1 ultrapure water (Milli-Q Direct, Milliore). The solution was 100x diluted prior ICP-MS analysis by solution containing 3% HNO3 and 0.5% HCl.

1.8 Scanning electron microscopy (SEM)

Scanning electron micrographs were taken with a Bruker Quantax400 EDS microscope equipped with a digital camera. The samples of the crystals for SEM measurement were prepared by transferring a small amount of activated or dried samples on a carbon-coated holder. The samples were gold-coated before the visualization.

1.9 Microplate readers

Spheroid imaging and ATP assay evaluation were done using BioTek Cytation 5 Cell Imaging Multimode Reader (Agilent Technologies, Winooski, VT). Spheroid images were captured using a 4× objective across different focal planes (z-stacks). The multiple plane images were combined into a z-projected image using BioTek Gen5 software (Agilent Technologies). Image segmentation and analysis were also performed in Gen5. The size (diameter) of the spheroid and its projected area (i.e., the 2D footprint of the spheroid from the imaging angle) were used as the descriptors of the spheroid size.

The luminescence in the ATP assay was recorded as relative luminescence units (RLU) using a BioTek SynergyMX microplate reader (Agilent Technologies), with an integration time of 1 second per well.

2 Synthesis and characterization of Ru(III) complex (RuC)

RuC comprises a ruthenium(III) metal center coordinated with 4-methylpyridine and dimethyl sulfoxide (DMSO) as axial ligands, complemented by four chlorides situated in the equatorial plane (Scheme 1 of the manuscript). The synthesis of RuC was performed in accordance with a previously established protocol [5], albeit with slight adjustments. Specifically, 20 mg (45.6 µmol) of K^+[*trans*-$RuCl_4(DMSO)_2$] were dissolved in a mixture of acetonitrile (5 mL) and acetone (AC) (10 mL). The resulting solution was filtered, and 10.9 µL (111.4 µmol, 2.4 equiv.) of 4-picoline were introduced. The solution exhibited a discernible color change to yellow within minutes and was subsequently stirred for additional 20 minutes. The mixture was concentrated to a final volume of 1 mL, and the product was precipitated by the addition of diethyl ether (DEE). The resulting precipitate was subjected to an ice-water bath for 8 minutes, during which the product coalesced

into larger particles, subsequently separated by filtration, washed with DEE, and dried. The isolated product was obtained as a yellow powder in 51% yield (10.6 mg).

The synthesis of RuC was followed by ^{1}H-NMR spectroscopy and electrospray ionization mass spectrometry (ESI-MS). It is important to note that the chosen model compound RuC, characterized by its modest size, presents an advantageous feature for ^{1}H-NMR analysis, as the methyl group on the pyridine ligand facilitates spectroscopic tracking of this paramagnetic coordination species. Still, in the context of paramagnetic compounds, conducting NMR spectroscopy encounters formidable challenges due to signal broadening effects, signal extreme chemical shifts, or even signal disappearance. Figure S1 demonstrates broadening of signals arisen from rapid relaxation processes, with observed chemical shift perturbations attributable to the influence of unpaired electrons. This perturbation, observable primarily in the axial ligands, manifests as a chemical shift of -15 ppm for DMSO. The signal of H2 was not observed because of paramagnetic effect of Ru(III) center. The signals at H3 and $-CH_3$ (H5) exhibit significant shifts to about -3 ppm, though not as pronounced. Furthermore, Figure S2 provides the ESI-MS spectrum of RuC, revealing solely the presence of the $[RuCl_4]^-$ peak. The absence of a molecular peak can be attributed to fragmentation processes inherent to the analytical methodology.

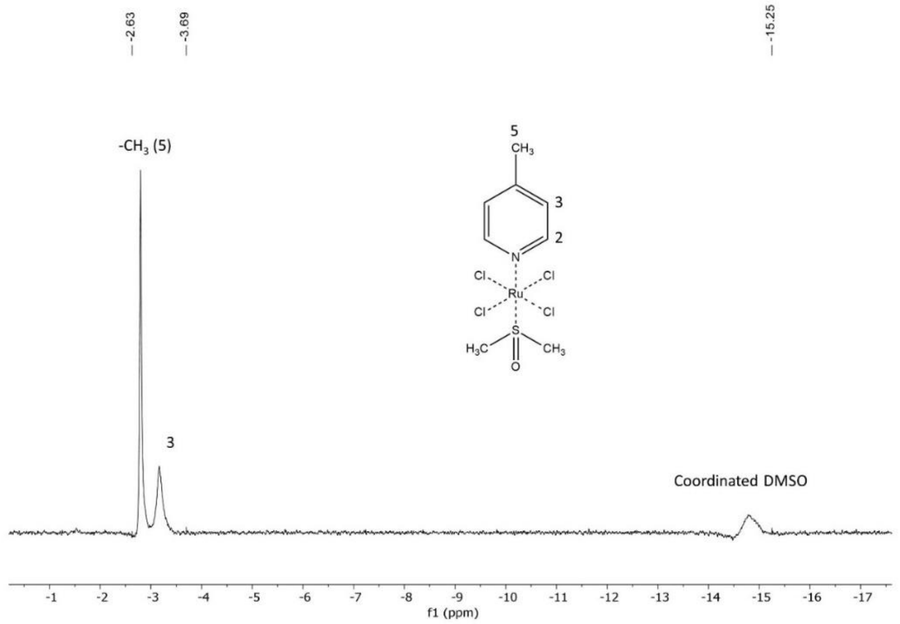

Figure S1. ^{1}H-NMR spectrum of RuC (700 MHz, D_2O, 298.2 K).

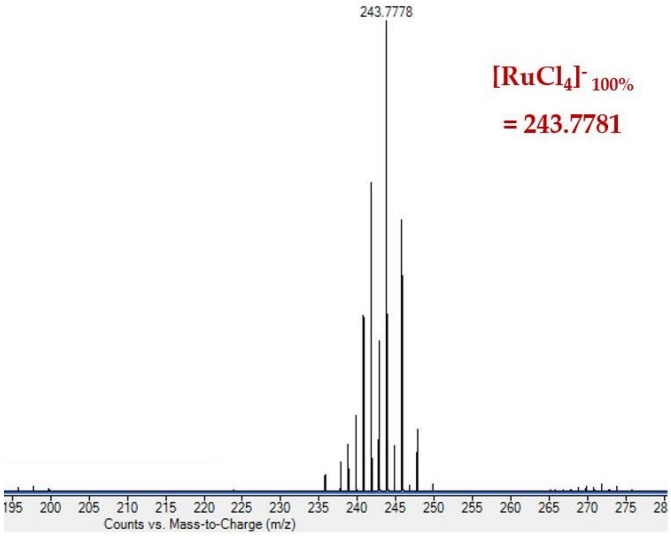

Figure S2. ESI-MS spectrum of RuC in negative mode. Experimental *m/z*: 243.7781, theoretical *m/z*: 243.7768 for $[RuCl_4]^-$ molecular fragment.

3 Crystal-to-crystal transformation

3.1 Single crystal X-Ray diffraction (SC-XRD)

K^+ ions, the coordinated lattice water molecules, and the $RuCl_4$ were highly disordered. For K^+ ions, some of them were found coordinating with the hydroxymethyl-O and the adjacent pyran-O of the γ-cyclodextrin (CD) and bridging the CD molecules to form a two-dimensional K-CD layer. Other K^+ ions were distributed in between the CDs as hydrated ions via hydrogen bonds. All K^+ were disordered, and the occupancies were fixed according to the electron densities. One of the hydroxymethyl groups was deprotonated to balance the charge of K^+. Occupancies of the disordered water molecules including the coordinated and lattice water were also fixed according to the difference Fourier map. The coordinated water molecules were not protonated, but the hydrogen atoms were considered and included in the formula. The $RuCl_4$ molecules were trapped in the cavity of CD. They were rotating because of no significant interaction with CD. Occupancies of the Ru were assigned at 0.40, 0.36, and 0.24, respectively, according to the difference Fourier maps. Each of the surrounding chlorine atoms were split over more than one position with the sum of the occupancies equal to each center Ru atom. In addition, there is some space with flat residual densities in the structure. The electron densities were masked via the "MASK" function in OLEX2. The relevant contents were considered and added to the whole formula of the structure. "ISOR" commends were used to restrain the displacement parameters of some disordered K and Cl atoms. Geometry restraint commends including "DFIX" and/or "DANG" were used for the H…O and H…H distances to make the hydrogen bonding more suitable. "TWIN" and "BASF" commends were used because of the slight twinning. The refined BASF value is 0.04(3).

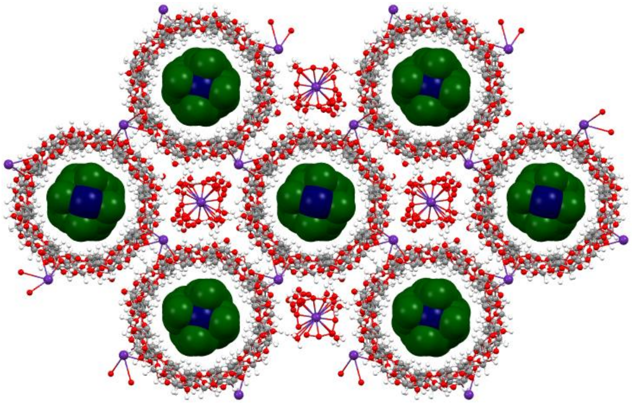

Figure S3. Crystal-to-crystal transformation of cubic crystal structure of CD-MOF-2 (Ru^+-based CD-MOF) into a columnar structure where the pores contain decomposition products of [$RuCl_4$(Py)(DMSO)] ($RuCl_4$ could be assigned). CCDC number 2420206.

4 Stability and morphology of crystals of CD-MOF-1

4.1 Powder X-Ray diffraction (PXRD)

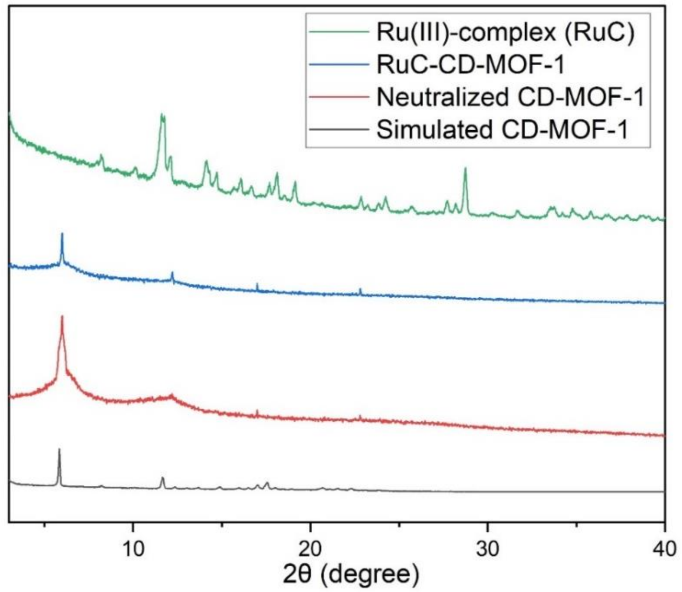

Figure S4. Comparison of PXRD patterns of RuC, drug loaded RuC-CD-MOF-1, post-synthesis processed, neutralized CD-MOF-1 and simulated pattern of CD-MOF-1 from SC-XRD data.

4.2 Crystal morphology

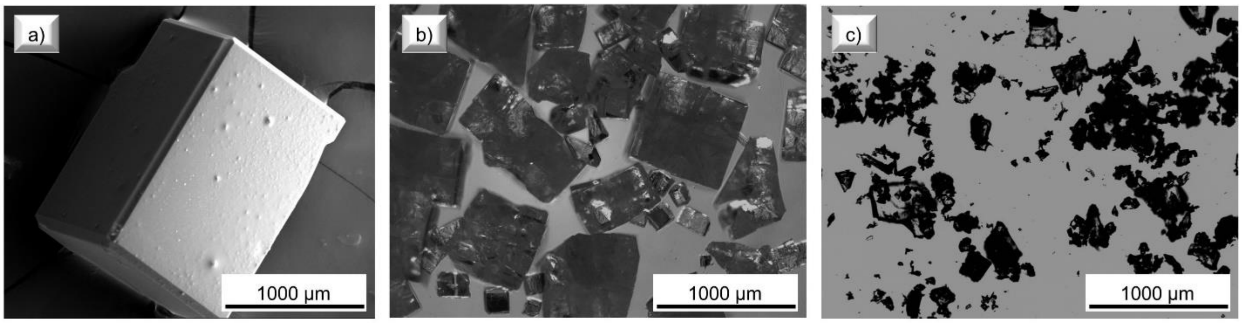

Figure S5. Crystals of a) processed and activated CD-MOF-1 (SEM), b) RuC-loaded CD-MOF-1 (optical microscope), c) crushed crystals of RuC-CD-MOF-1 as application form for toxicity assessment (optical microscope).

5 Stability studies of RuC

Incorporating small organic molecules into the crystals of CD-MOF-1 crystal typically takes 3 to 5 days [6]. Nevertheless, the Ru(III) complexes are known for their low stability. Previous studies have shown that these complexes can break down relatively quickly through processes like hydrolysis or solvolysis [7]. Scheme S1 provides a depiction of the conceivable hydro-/solvolytic products of RuC complex (excluding their possible combinations, which is further complicating their exact determination and structural characterization). Therefore, the shortest possible adsorption period and proper adjustment of experimental environment and conditions are the key to successful and efficient RuC incorporation inside CD-MOF.

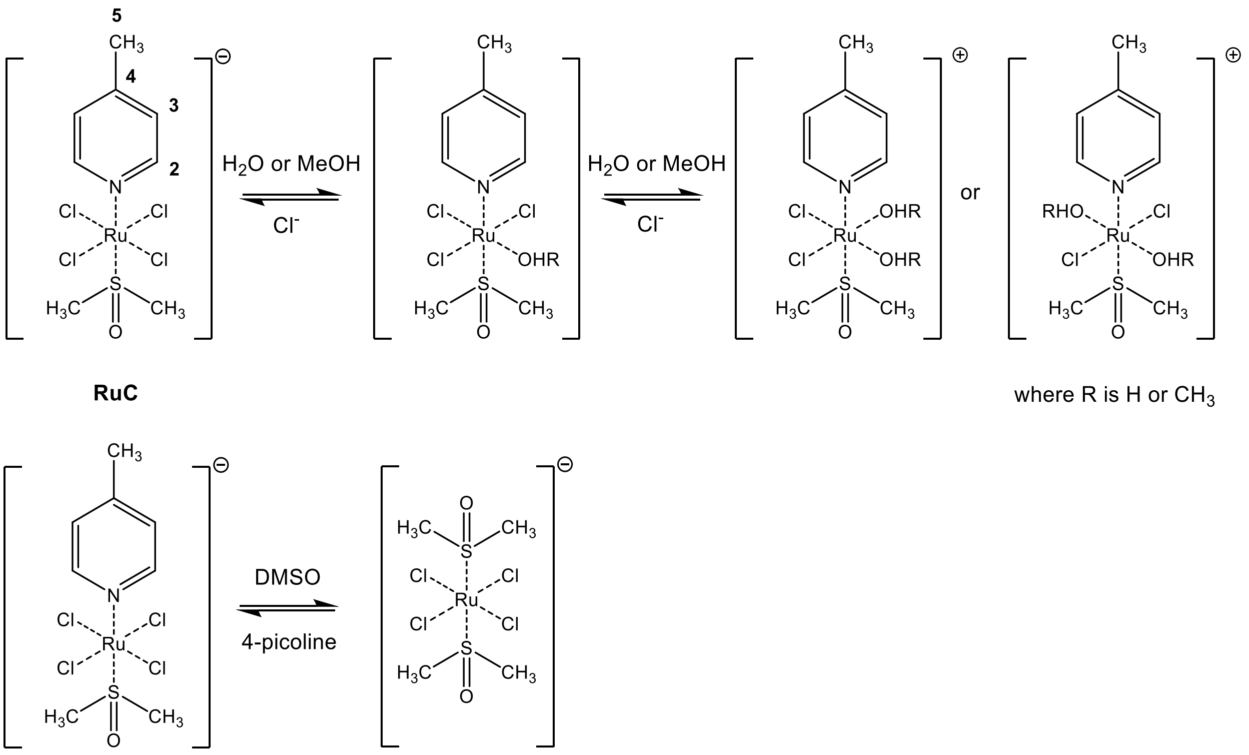

Scheme S1. Some hydro-/solvolytic products of RuC (excluding their possible combinations).

Pursuing the identification of optimal conditions for enhancing the stability of RuC, a series of experiments were conducted, employing diverse solvents, pH, and an excess of chloride ions to mitigate the displacement of Cl^- ligands by H_2O in equatorial position. A summary of experiments conducted is presented in Table S1, while the corresponding spectra can be found in Figure S6-S13. While the precise characterization and assignment of distinct complex forms remains elusive and was not a goal of this study, their occurrence or the preservation of RuC's stability constitutes a pivotal facet of this investigation helping us to proceed with the most suitable conditions for drug loading study.

Table S1. Experimental conditions in stability experiments performed.

Experiment no.	Solvent	Addition	pH	Forms of Ru(III) complex	^{1}H NMR spectrum in Fig.
1	CD_3OD	-	-	3	S4
2	CD_3OD	KCl	-	3	S5
3	CD_3OD	γ-CD	-	3	S6
4	CD_3OD	1M DCl	4	3	S7
5	CD_3OD	1M DCl	2	3	S8
6	DMSO-d_6	1M DCl	6	1	S9
7	DMSO-d_6	1M DCl	4	1	S10
8	DMSO-d_6	1M DCl, γ-CD	4	1	S11

The initial experiment was conducted using CD_3OD as the solvent and the experiment was followed using ^{1}H NMR spectroscopy checking right after mixing (5 min), after 24, and 48 h (Figure S6). The spectra show two distinct signals denoted to H2 and -CH_3 (H5) with integration 2:3 to each other at -2.2 ppm and -2.6 ppm, respectively, and a broad signal for coordinated DMSO at about -13.2 ppm. Notably, after 24 h, there are new sets of H3 and H5 signals appearing corresponding to degradation products, which grow more intense by time to the prejudice of the original signals of RuC. This observation is further substantiated by the appearance of a secondary signal(s) attributed to coordinated DMSO. Following the RuC transformation for 48 h, RuC degradation becomes more evident as can be observed by a substantial reduction in the intensity of the original signals and increase of new ones. Notably, no signals corresponding to free 4-methylpyridine (7-9 ppm) were detected.

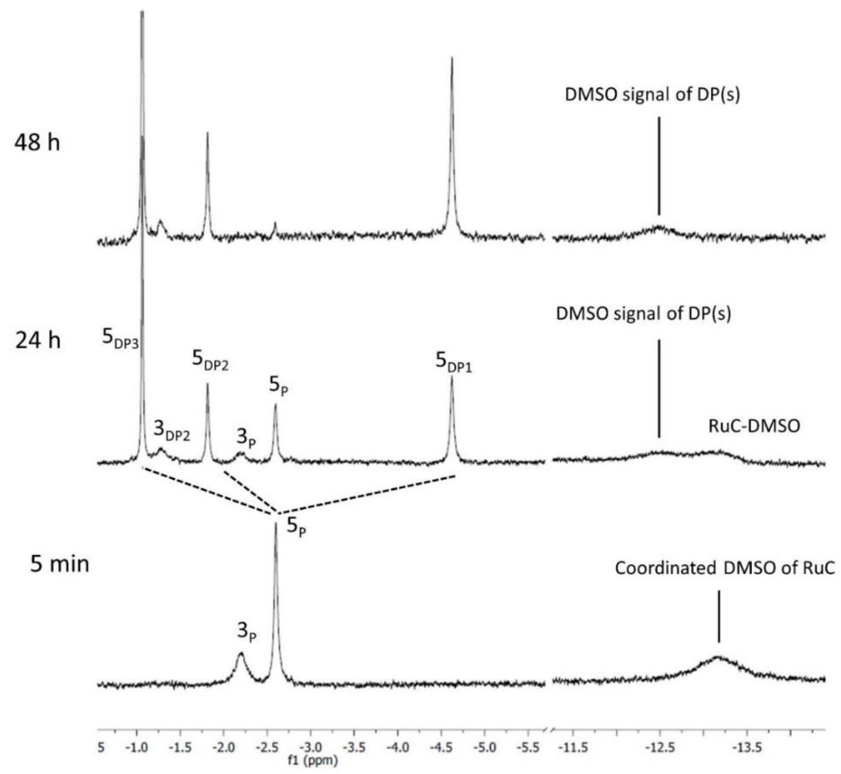

Figure S6. ^{1}H NMR spectrum of RuC (hydrogen signals of product RuC marked by number and P) right after dissolution in CD_3OD, after 24 h and 48 h (600 MHz, 298.2 K). The formation of three degradation products (DP1-DP3) of RuC complex can be observed.

In the next experiment, a 10-equimolar excess of KCl was introduced to the CD_3OD solution containing RuC and the mixture was checked by ^{1}H-NMR spectroscopy at 72 and 120 h (Fig. S7). Nevertheless, a recurrent pattern emerged, as the formation of degradation products transpired in a manner consistent with previous observations.

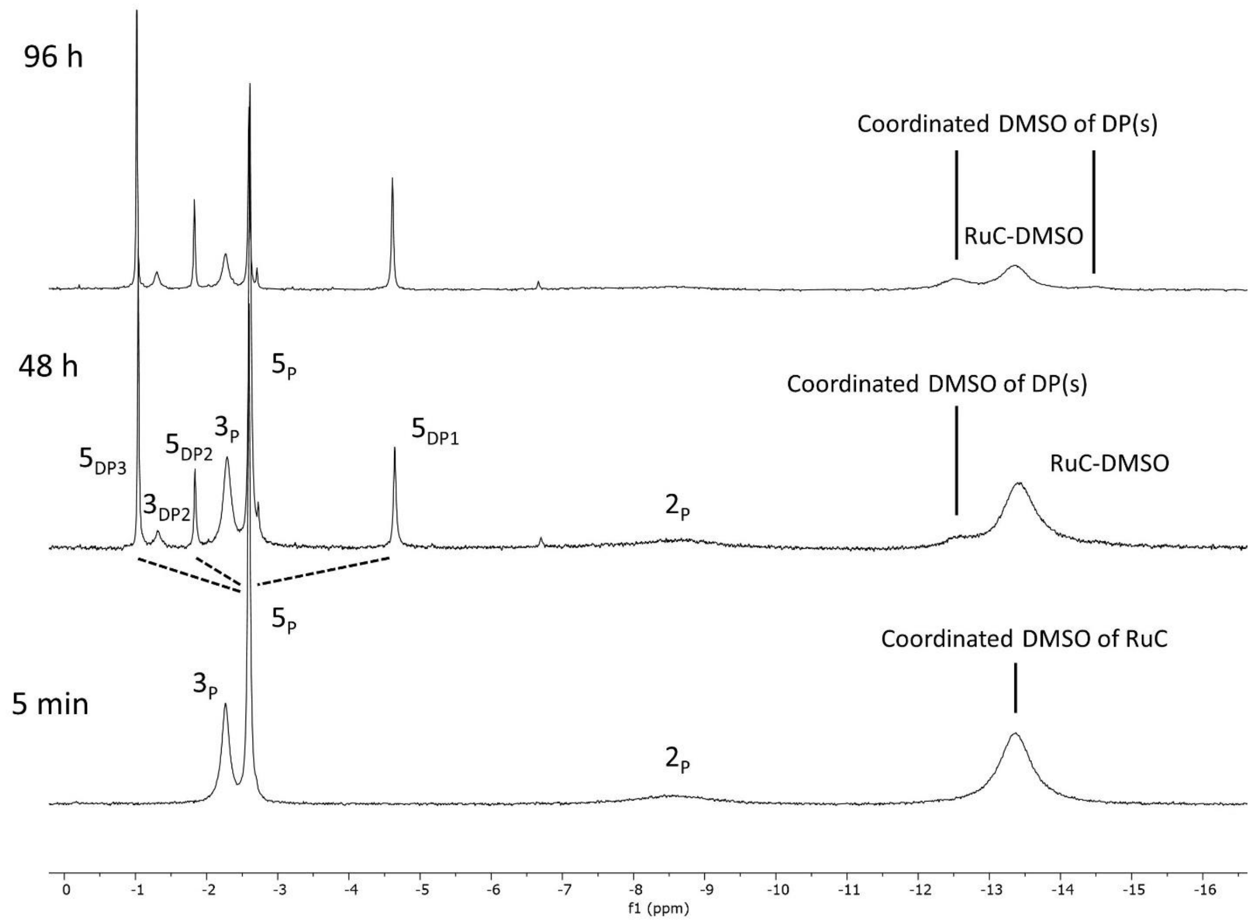

Figure S7. ^{1}H-NMR spectra of RuC in CD_3OD with addition of 10 equiv. of KCl (700 MHz, 298.2K). Hydrogen signals of product RuC marked by number and P, formation of three degradation products (DP1-DP3) of RuC complex can be observed.

In the subsequent experiment, γ-cyclodextrin (γ-CD) was introduced into the CD_3OD solution containing RuC, with the intent of harnessing host-guest complexation as a mean of enhancing complex stability. ^{1}H NMR spectrum was acquired immediately after the preparation and on 3rd and 5th day of the experiment (Fig. S8). However, the introduction of γ-CD yielded results mirroring prior findings.

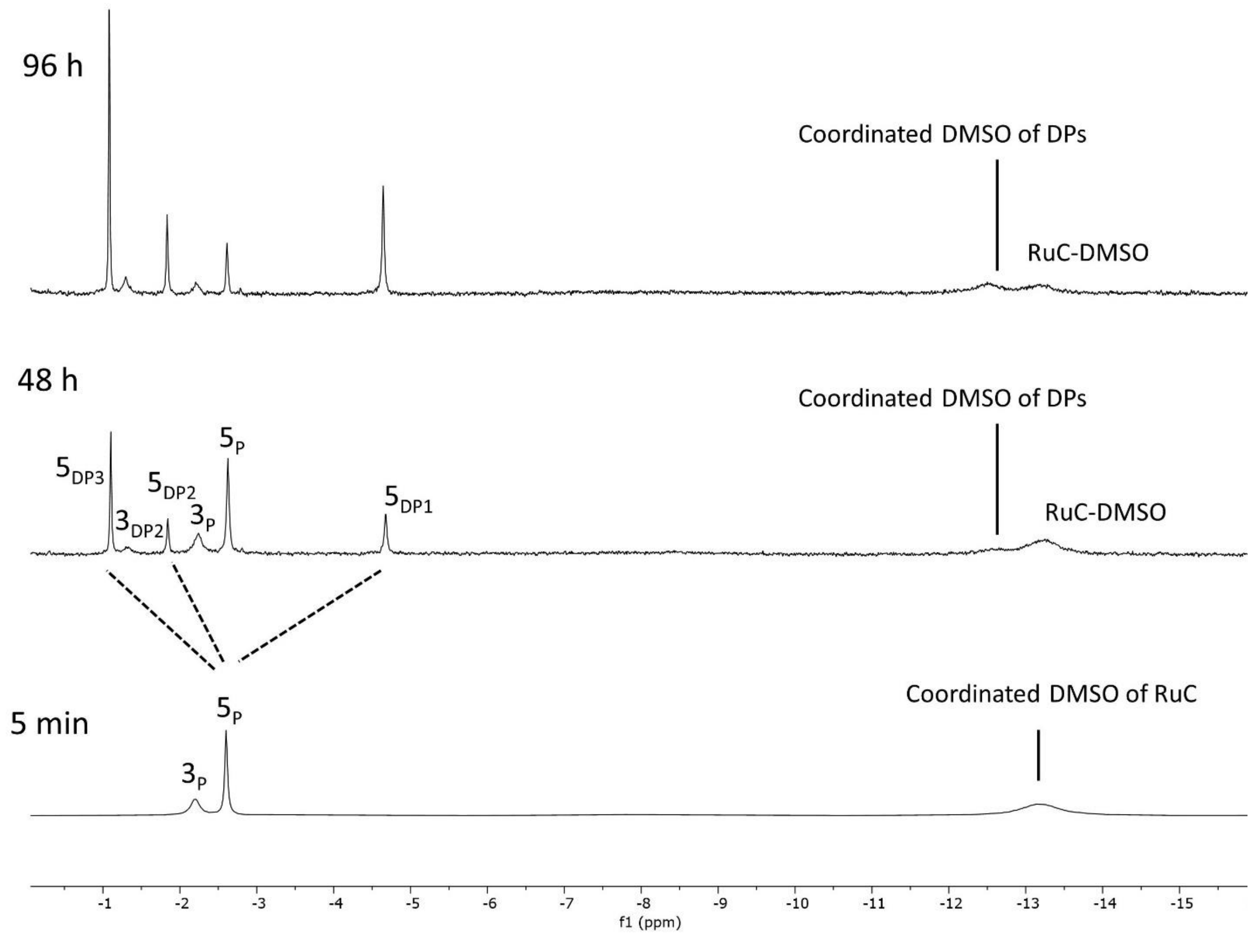

Figure S8. ^{1}H-NMR spectra of RuC in CD_3OD with addition of 2 equiv. of γ-CD (700 MHz, 298.2K). Hydrogen signals of product RuC marked by number and P, formation of three degradation products (DP1-DP3) of RuC complex can be observed.

Considering the unsuccessful attempts to enhance RuC stability analogous studies have been suggested exploring potential benefits in modulating pH levels. The pH of the RuC solution in its native state is approximately 6. Additional stability experiments were conducted at pH 2 and 4, necessitating the introduction of 1 M hydrochloric acid (HCl or its deuterated form) in precise quantities. The alteration in pH was monitored using a pH meter.

The ^{1}H NMR measurement of the CD_3OD solution of RuC (Fig. S9) provides a chronological record taken immediately after its preparation, at 40 minutes, one, two, three, and four hours, and subsequently after one, two, and three days. The data obtained in this experiment indicate that the hydrolysis process begins as early as one hour into the experiment.

Similarly, ^{1}H NMR measurement was conducted at identical time intervals using the RuC solution in CD_3OD at pH 2. The analysis of the data obtained reveals that the hydrolysis process is initiated after 2 h (Fig. S10). It is evident that the reduction of pH does not provide control over the hydrolytic process.

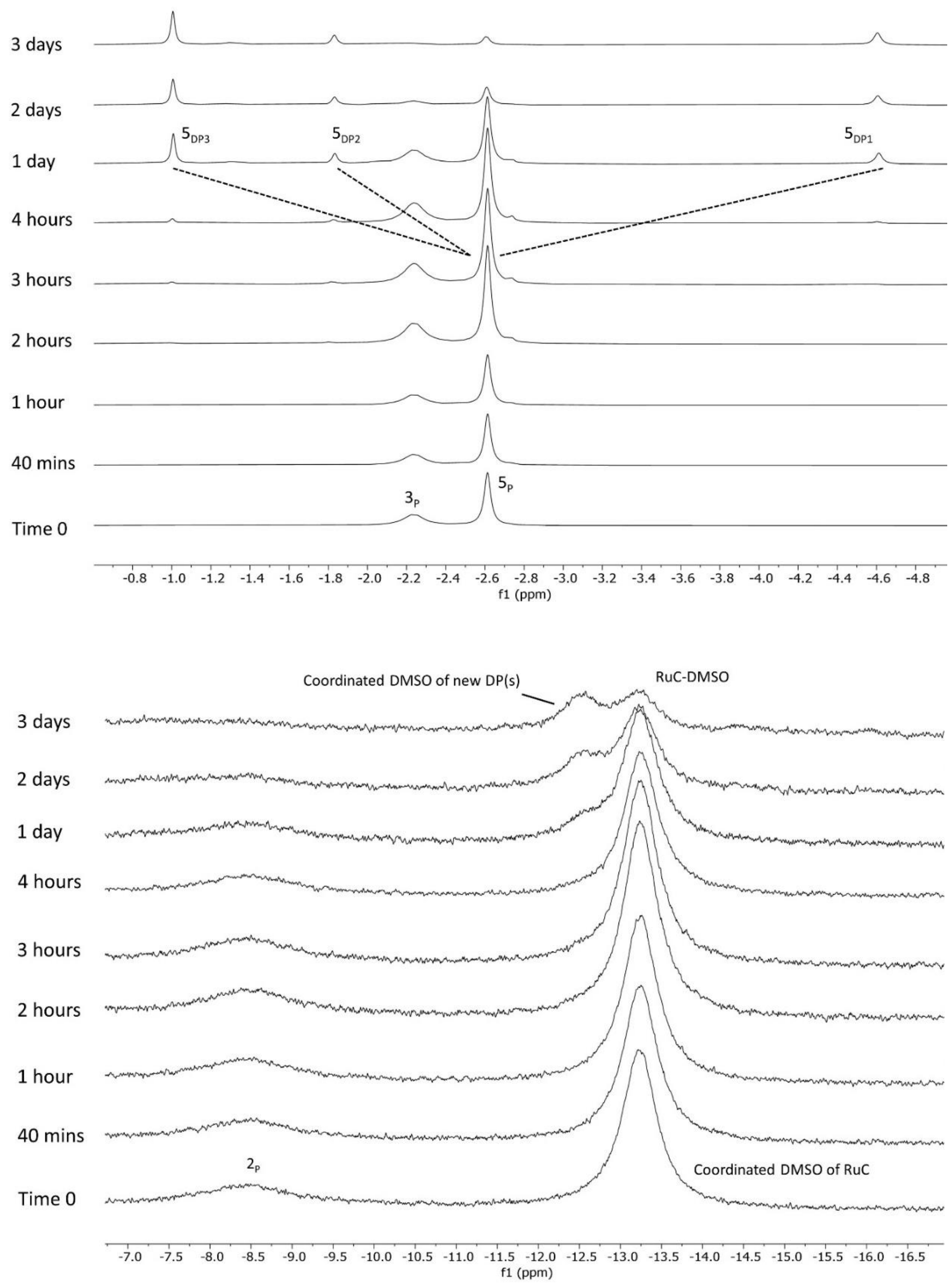

Figure S9. ^{1}H-NMR spectra of RuC in CD_3OD at pH = 4 (600 MHz, 298.2K). Hydrogen signals of product RuC marked by number and P, formation of at least three degradation products (DP1-DP3) of RuC complex can be observed.

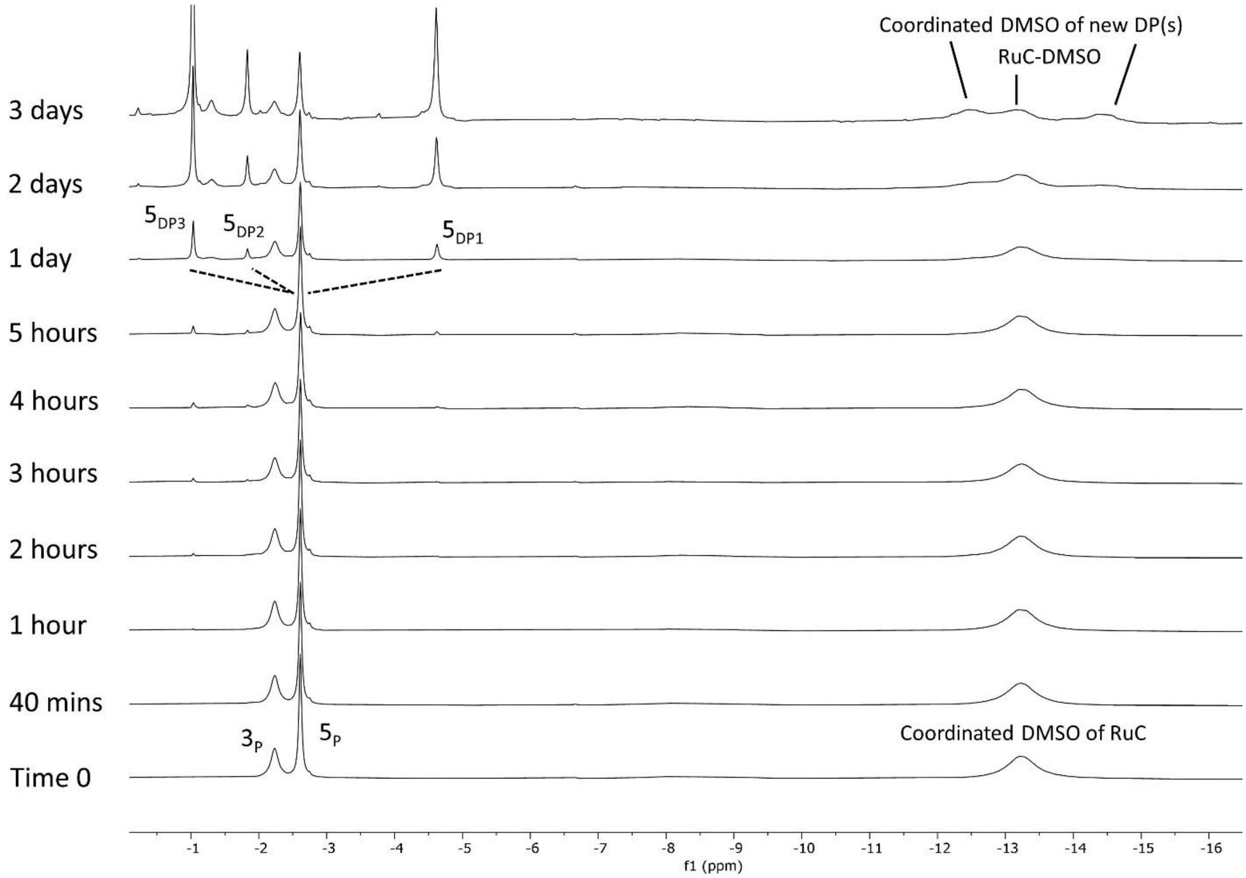

Figure S10. ^{1}H-NMR spectra of RuC in CD_3OD at pH = 2 (600 MHz, 298.2K). Hydrogen signals of product RuC marked by number and P, formation of at least three degradation products (DP1-DP3) of RuC complex can be observed. Signals of free 4-methylpyridine could be also observed after 4 h.

In the next step of optimization, we tested DMSO-d_6 as solvent. The amount and number of degradation products was limited even after 4 days, after which signals of free 4-picoline appeared (Fig. S11). However, further steps were taken to further mitigate solvolytic processes occurring at the axial coordination position (Scheme S1).

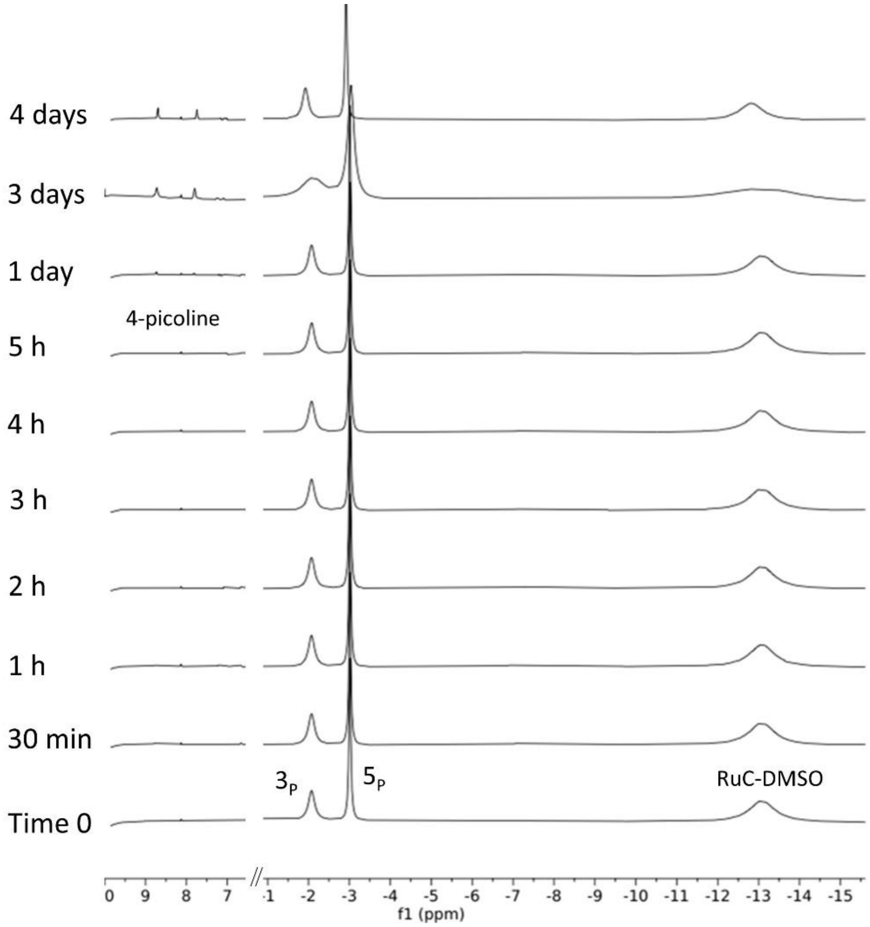

Figure S11. ^{1}H NMR spectra of RuC in DMSO-d_6 at pH 6 (700 MHz, 298.2K).

The experiment in DMSO-d_6 at pH 4 revealed similar results (Fig. S12). Subsequently, the experiment was revisited using RuC in DMSO-d_6 at pH 4, supplemented with γ-CD to leverage host-guest complexation for additional stabilization (Fig. S13).

A comparative analysis of the DMSO-d_6 experiments leads to the conclusion that the additional stabilization of RuC was not achieved, but as such it is sufficient for the drug loading study. In summary, it was determined that the utilization of DMSO as the solvent at pH 4 represents the optimal condition for RuC stabilization in solution leading to stability for up to four days.

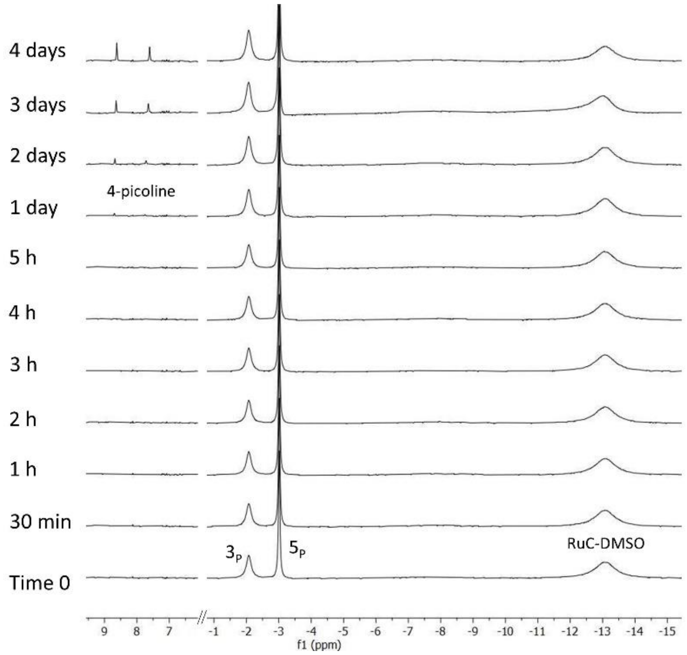

Figure S12. ^{1}H NMR spectra of RuC in DMSO-d_6 at pH 4 (700 MHz, 298.2 K).

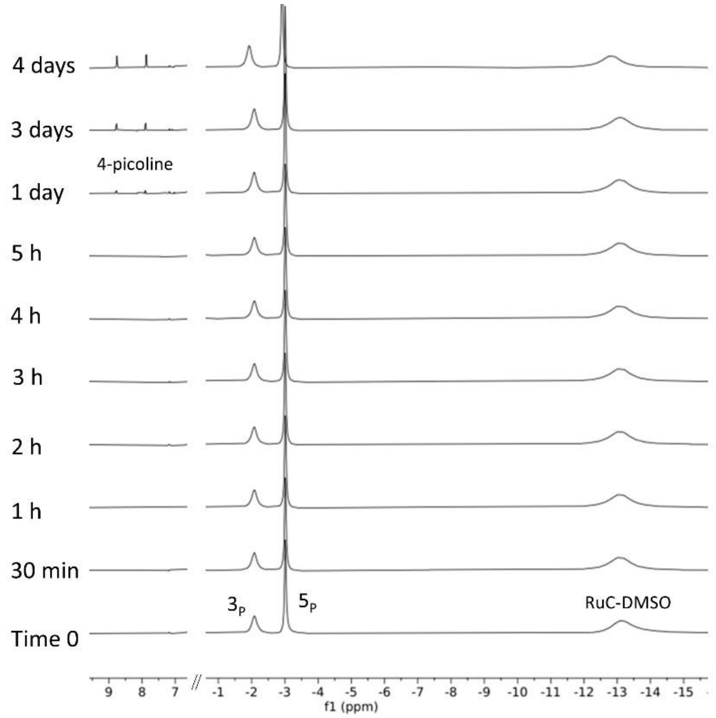

Figure S13. ^{1}H NMR spectra of RuC in DMSO-d_6 at pH 4 with addition of 1 equiv. of γ-CD (700 MHz, 298.2 K).

6 Drug loading studies

6.1 ^{1}H NMR spectroscopic study

The orange crystals (2 mg) were dissolved in DMSO-d_6 (450 μL) under vigorous sonication and ^{1}H NMR spectrum was measured showing signals for RuC, γ-CD, and a small amount of free 4-methylpyridine (Fig. S14). Since the CH_3 (H5) signal of RuC is the least affected by the Ru(III) center, its integration was compared with the signal of H1' of γ-CD showing approximately 3:4 ratio (2.4 mg of RuC per 10 mg of CD-MOF-1, 24 wt% RuC).

The ^{1}H NMR spectroscopy is burdened by lower sensitivity but also by signal broadening because of the paramagnetic effect of Ru(III) (unreliable integration of RuC methyl signal but also of cyclodextrin from which the values were calculated).

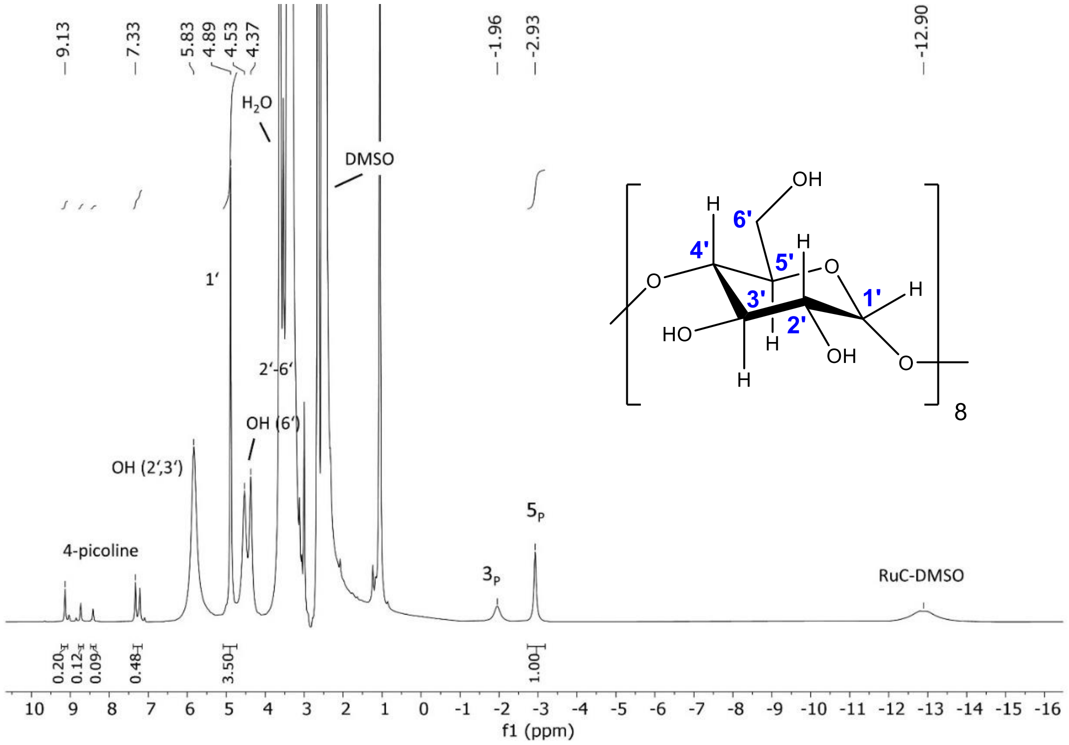

Figure S14. ^{1}H NMR spectrum of dissolved RuC-loaded CD-MOF-1 crystals in DMSO-d_6 (pH 4, 700 MHz, 298.2 K). The ratio between RuC and γ-CD is 3:4.

6.2 UV-Vis spectroscopic study

The RuC sample was measured using UV-Vis spectroscopy across a concentration range from 20 to 100 μM (in DMSO, pH 4) to construct a calibration curve (Fig. S15). Subsequently, UV-Vis spectra were acquired for samples before and after RuC loading into crystals of CD-MOF-1. It was determined that the residual concentration of RuC in the solution after its adsorption decreased from the original 90 μM to 68.25 μM. This reduction can be attributed to the effective entrapment of RuC inside CD-MOF-1 crystals. Using fitting in the calibration curve, the amount of absorbed RuC corresponds to 21.75 μM, *i.e.*, 1.4 mg of RuC per 10.0 mg of CD-MOF-1 (14 wt%), suggesting that the ratio between RuC and γ-CD of the CD-MOF-1 is approximately 1:2 (γ-CD concentration could be obtained from the empirical formula $[(C_{48}H_{80}O_{40})(KOH)_2]_n$ of CD-MOF-1). M_W (g/mol): Ru - 101.9; RuC - 451.82; CD-MOF-1 $[(C_{48}H_{80}O_{40})(KOH)_2]$ - 1409.33.

The value obtained using UV-vis spectroscopic measurement is determined indirectly. It characterizes the residual content of RuC in the solution, *i.e.*, the actual RuC content in crystals of CD-MOF-1 as applied to cell culture is likely lower, as some amount of RuC was lost/leached out during crystal processing (the crystals were filtered off, washed several times, and dried before their biological application).

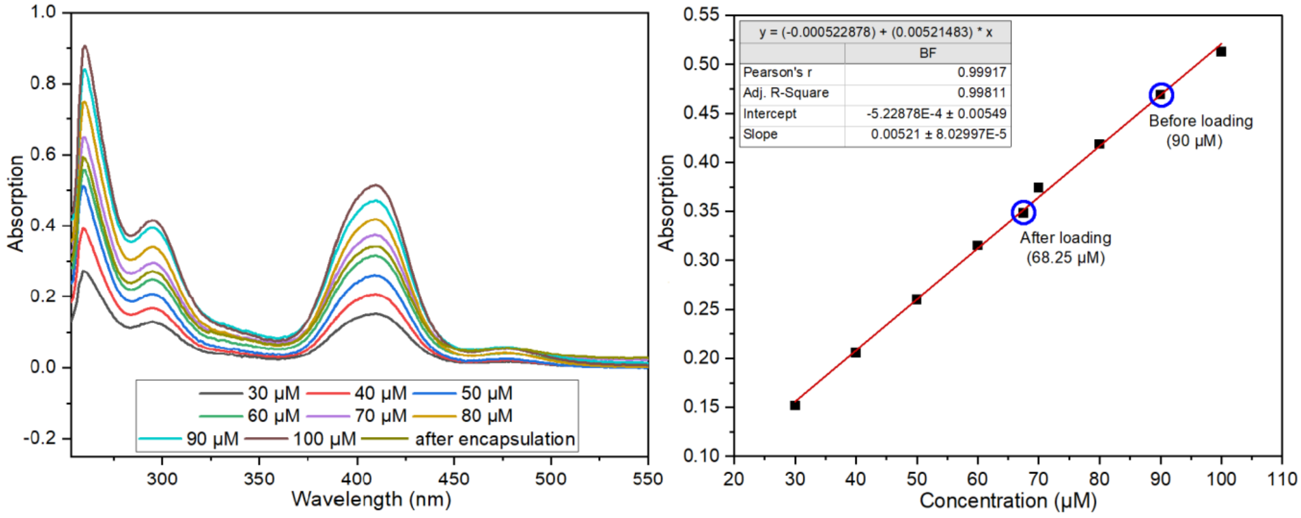

Figure S15. UV-Vis calibration curve of RuC and fitting of drug loading data.

7 HepG2 cell cultivation and spheroid preparation

The cell culture and spheroid experiments were conducted as previously reported [8, 9]. Briefly, HepG2 cells were grown in low glucose (1 g/L) MEM with phenol red, supplemented with 1% (v/v) MEM non-essential amino acids, 1 mM sodium pyruvate, and 1.5 g/L sodium bicarbonate. The medium was supplemented with 10 % of fetal bovine serum and sterile-filtered through 0.2 µm PES filter. The cells were routinely cultured in monolayer cultures in 25 cm² cell culture flasks kept at 37 °C in a 5 % CO_2 humidified atmosphere. The cells were passaged into new flasks using trypsin/EDTA twice per week before reaching 80 % confluency. For the spheroid assays, the concentration of the trypsinized cell suspension was determined using the automated cell counter Cellometer™ (Nexcelom Bioscience, Lawrence, MA) and adjusted to 5,000 cells/mL with fresh culture medium.

The inner (6 x 10) wells of black 96-well plates were coated with 50 µL of melted sterile 1.5 % (w/v) agarose in 0.9 % (w/v) NaCl solution. After the agarose solidified, 200 µL of the cell

suspension (~1000 HepG2 cells) was pipetted per well, except the assay blank wells filled with 200 µL of culture medium without cells. Agarose prevents cell attachment to the microplate bottom. Its concave shape causes HepG2 cells to aggregate in the center, promoting the formation and 3D growth of a single spheroid per well. The peripheral wells were filled with 250 µL of sterile phosphate-buffered saline (PBS). The plates were cultured at 37 °C in 5 % CO_2 for 4 days prior to exposure. The formation, size and morphology of the spheroids was documented by brightfield imaging using BioTek Cytation 5. The average size of the spheroids prior the exposure was 311 ± 3 µm, corresponding to the projected area of 76187 ± 1170 μm^2.

8 HepG2 spheroid exposure and toxicity assessment

Stock solutions of each experimental treatment (γ-CD, CD-MOF-1, RuC, and RuC-CD-MOF-1) were prepared in 10% DMSO (v/v) at a concentration 50X of the desired final concentration. The stock solutions, or 10% DMSO (v/v) as the control, were aseptically diluted in sterile culture medium supplemented with antibiotics (P/S) and added to the spheroids by replacing 100 µL of the old culture media in each microplate well. The final concentration of DMSO in all treatment wells, including solvent control wells, was 0.2% (v/v). The tested concentrations of γ-CD and CD-MOF-1 were 5, 25, 50, 100, and 250 µg/mL. RuC concentrations were set at 100, 200, 300, 400, and 500 µg/mL for both naked RuC and encapsulated RuC-CD-MOF-1 treatments. The RuC content in CD-MOF-1, determined by UV-Vis spectroscopy (14 wt%), was used to calculate the required concentrations of RuC-CD-MOF-1 to achieve the desired RuC levels. Each experimental treatment was pipetted into the microplate in three technical replicates.

After the exposure initiation, the spheroid plates were incubated at 37 °C in 5 % CO_2 humidified atmosphere for 2 weeks. On the 7th day of exposure, 100 µL of medium in each well was replaced with fresh culture medium (without the tested compounds), and the experiment was continued for

an additional 7 days. The spheroid growth and morphology during the experiment were documented by BioTek Cytation 5. The representative spheroid images are presented in Figures S17, S18, S20, and S21.

During the 14 days of exposure, the control spheroids exhibited continuous growth, with the average diameter of the control spheroids increasing to 503±11 μm and the projected area to 199229 ± 8329 μm^2, which represents 261% of the initial spheroid area. The results for spheroid growth were reported as a percentage of the initial spheroid area, i.e., the area of a spheroid at a given time point relative to its initial area at the beginning of the exposure on day 4 (set as 100%).

After the last spheroid imaging, the number of viable cells in the spheroids was determined by assessment of ATP content in the culture using CellTiter-Glo® 3D Cell Viability Assay. The spheroids and also blank wells (containing agarose and culture medium without the cells) were rinsed two times with PBS (100 μL of media per wash were removed and replaced with PBS in each step). After the last rinse, 100 μL of PBS was removed and replaced with 100 μL of CellTiter-Glo® 3D Reagent, which facilitates the release of ATP from viable cells and contains components for an ATP-dependent bioluminescent reaction. The microplate was then vigorously stirred for 5 min on a microplate shaker and incubated for another 25 min in dark at room temperature. The luminescence (RLU) was recorded using the BioTek SynergyMX reader. The average luminescence readouts from the assay blank wells were subtracted from all measured values in the plate. Blank-subtracted values of each well were then compared to the averaged blank-subtracted luminescence value of the control wells and expressed as % of the control.

Each experiment, with all treatments conducted in triplicate wells, was carried out three times independently, i.e. in microplates with independently prepared spheroids. Values produced from independent experiments (n=3) were used for statistical data analysis. Data visualization and

response modeling were conducted using GraphPad Prism 10. Dose-response modelling for ATP was done using a non-linear regression with inhibitor vs. response model (variable slope, four-parameter). Dose-dependent changes in spheroid area were modeled using a centered fourth-order polynomial fit. Time-dependent changes of spheroid area were modeled using a non-linear regression agonist vs. response model (three parameter), except RuC and RuC-CD-MOF-1 at both 400 µg/mL and 500 µg/mL concentrations modeled using centered fourth-order polynomial fit. Statistically significant ($P<0.05$) differences between the treatments and the control were determined using SigmaPlot 15.0 by a one-way analysis of variance (ANOVA) followed by Dunnett‘s post hoc test. In cases where the data were not normally distributed or did not have equal variances, a non-parametric Kruskal-Wallis ANOVA on ranks was used (no significant differences were found in these instances).

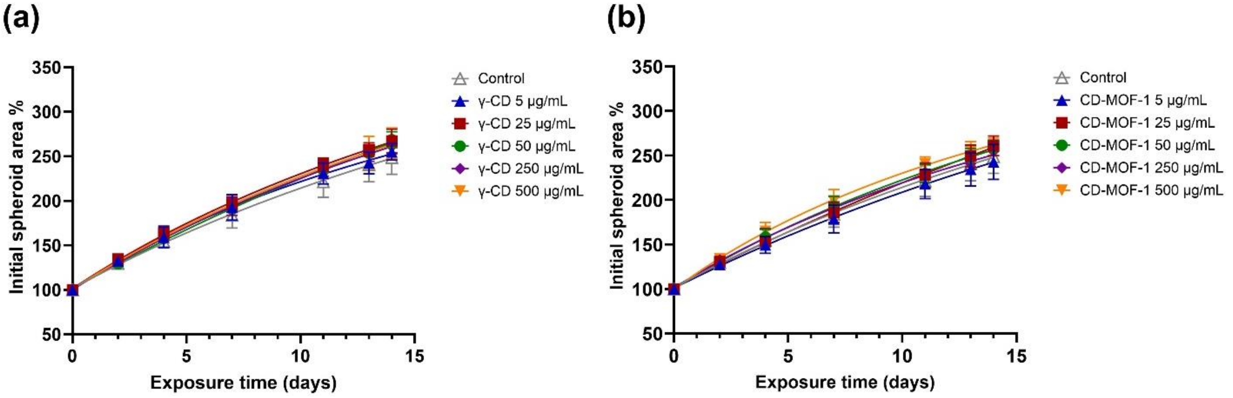

Figure S16. Monitoring of the HepG2 spheroid growth during 2-week exposure. Plots are based on the relative change in the projected spheroid area over time under a given treatment, compared to the initial area at the start of the exposure (day 0, set as 100%). Samples of: a) γ-CD, b) CD-MOF-1. No treatment was significantly different from the solvent control (ANOVA, $P<0.05$).

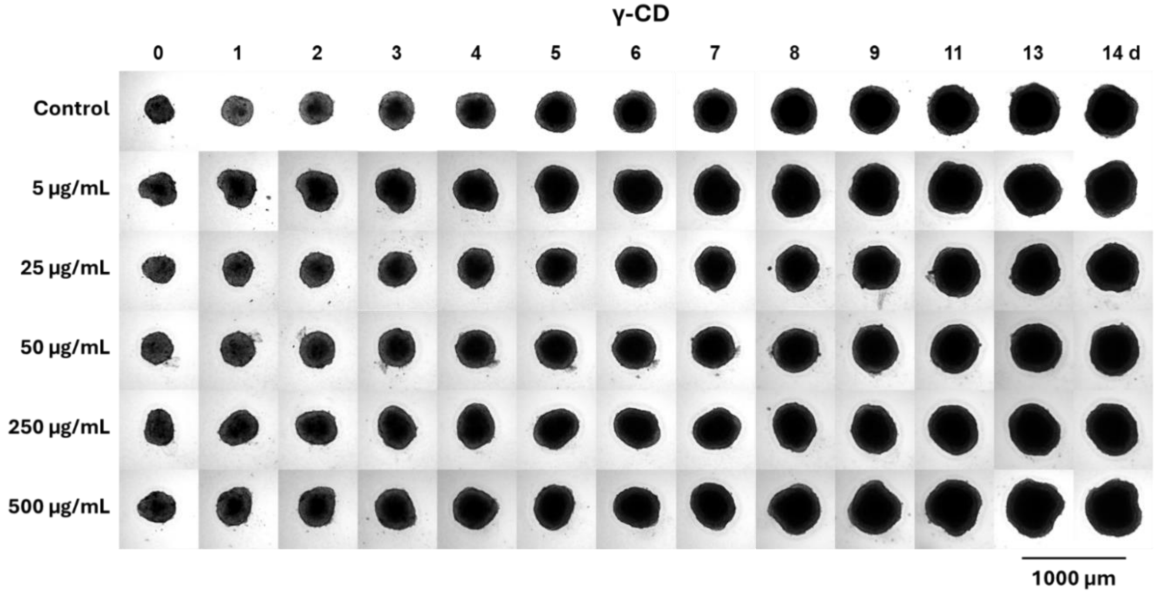

Figure S17. Representative Z-projected images of HepG2 spheroids treated over 14 days with various concentrations of γ-CD.

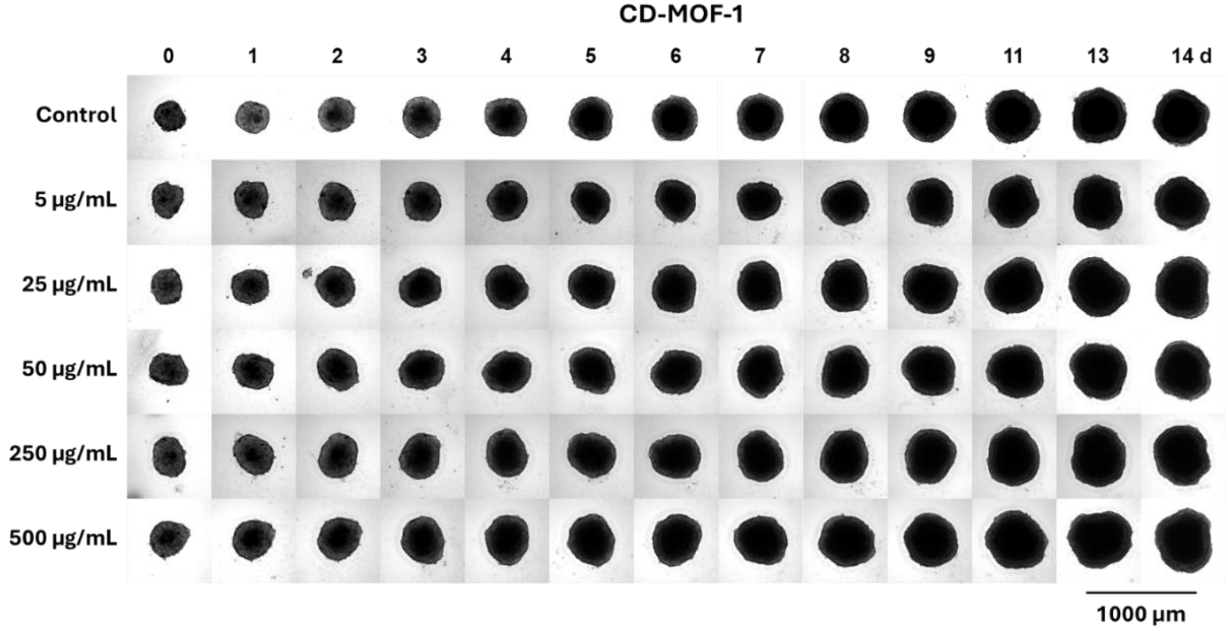

Figure S18. Representative Z-projected images of HepG2 spheroids treated over 14 days with various concentrations of CD-MOF-1.

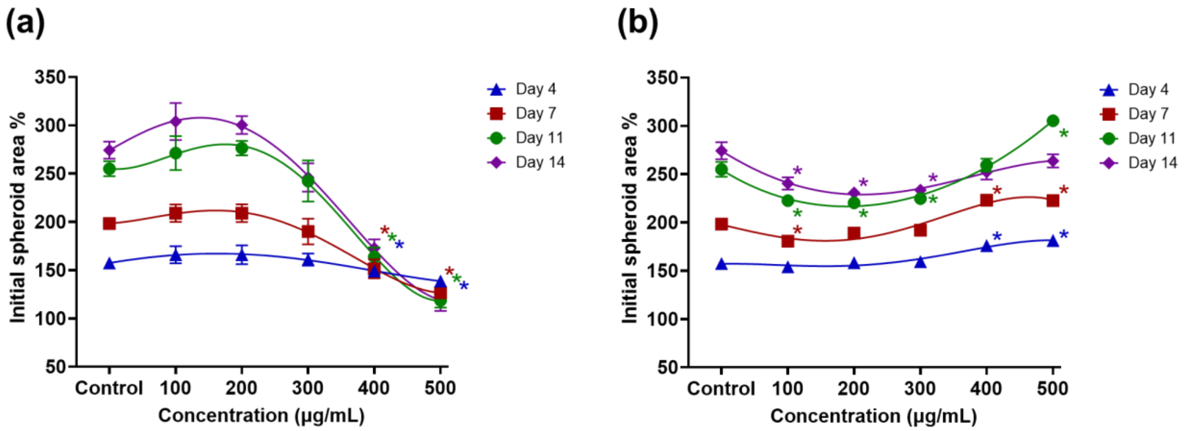

Figure S19. Dose-response of the HepG2 spheroid growth during 2-week exposure to naked and encapsulated RuC. Plots are based on the relative change in the projected spheroid area over time under a given treatment, compared to the initial area at the start of the exposure (day 0, set as 100%). Samples of: a) RuC, and b) RuC-CD-MOF-1 (the concentrations refer to the encapsulated RuC in the test system). Data represent means±SEM from independently repeated experiments (n=3). Asterisks represent treatments significantly different from the solvent control at the given time point (ANOVA, $P<0.05$).

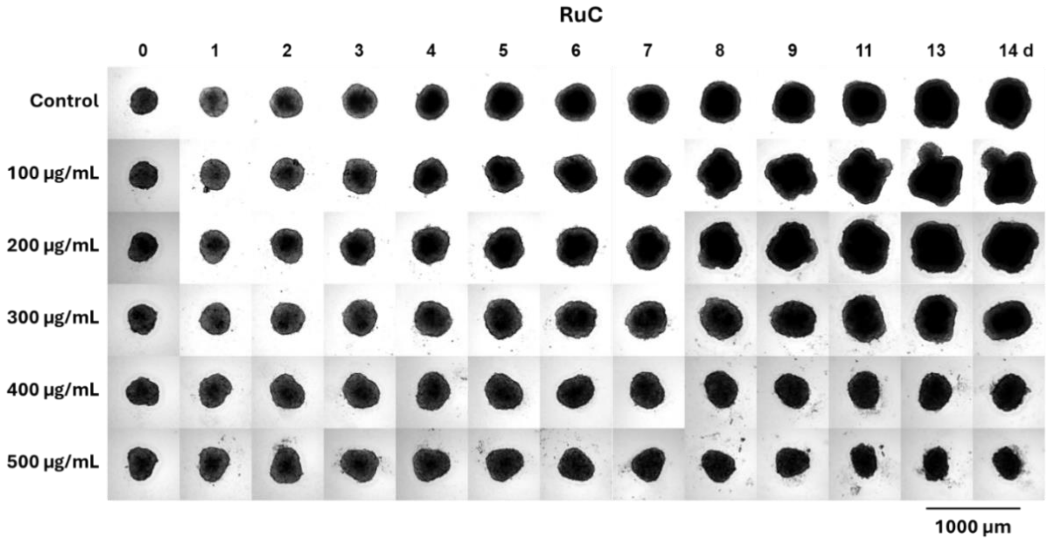

Figure S20. Representative Z-projected images of HepG2 spheroids treated over 14 days with various concentrations of RuC.

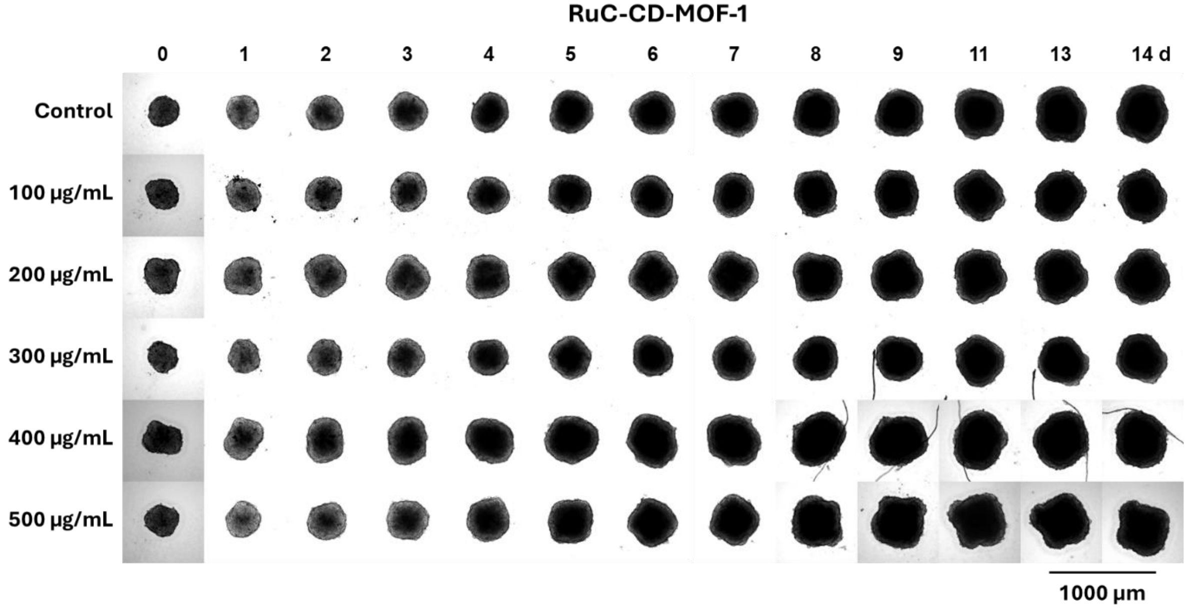

Figure S21. Representative Z-projected images of HepG2 spheroids treated over 14 days with various concentrations of RuC-CD-MOF-1 (the concentrations refer to the encapsulated RuC in the test system).

9 References

[1] CrysAlisPro, Ver. 36.45; Rigaku Oxford Diffractometer Technologies: 2012.

[2] Sheldrick, G. M. A short history of SHELX. Acta Crystallogr., Sect. A: Found. Crystallogr. 2008, 64, 112−122.

[3] Sheldrick, G. M. Crystal structure refinement with SHELXL. Acta Crystallogr., Sect. C: Struct. Chem. 2015, 71, 3−8.

[4] Dolomanov, O. V.; Bourhis, L. J.; Gildea, R. J.; Howard, J. A. K.; Puschmann, H. OLEX2: a complete structure solution, refinement and analysis program. J. Appl. Crystallogr. 2009, 42, 339−341.

[5] Alessio, E., Balducci, G., Calligaris, M., Costa, G., Attia, W. M., & Mestroni, G. (1991). Synthesis, molecular structure, and chemical behavior of hydrogen trans-bis (dimethyl sulfoxide) tetrachlororuthenate (III) and mer-trichlorotris (dimethyl sulfoxide) ruthenium (III): the first fully characterized chloride-dimethyl sulfoxide-ruthenium (III) complexes. *Inorganic Chemistry*, *30*(4), 609-618.

[6] Hartlieb, K. J., Ferris, D. P., Holcroft, J. M., Kandela, I., Stern, C. L., Nassar, M. S., ... & Stoddart, J. F. (2017). Encapsulation of ibuprofen in CD-MOF and related bioavailability studies. *Molecular pharmaceutics*, *14*(5), 1831-1839.

[7] Bacac, M., Hotze, A. C., van der Schilden, K., Haasnoot, J. G., Pacor, S., Alessio, E., Sava, G., Reedijk, J. (2004). The hydrolysis of the anti-cancer ruthenium complex NAMI-A affects its DNA binding and antimetastatic activity: an NMR evaluation. *Journal of inorganic biochemistry, 98*(2), 402-412.

[8] Schneider, M., Grossi, M. F., Gadara, D., Spáčil, Z., Babica, P., & Bláha, L. (2022). Treatment of cylindrospermopsin by hydroxyl and sulfate radicals: Does degradation equal detoxification?. *Journal of Hazardous Materials*, *424*, 127447.

[9] Chowdhury, R. R., Grosso, M. F., Gadara, D. C., Spáčil, Z., Vidová, V., Sovadinová, I., & Babica, P. (2024). Cyanotoxin cylindrospermopsin disrupts lipid homeostasis and metabolism in a 3D in vitro model of the human liver. *Chemico-Biological Interactions*, *397*, 111046.
